# Comparative analysis of antibody- and lipid-based multiplexing methods for single-cell RNA-seq

**DOI:** 10.1186/s13059-022-02628-8

**Published:** 2022-02-16

**Authors:** Viacheslav Mylka, Irina Matetovici, Suresh Poovathingal, Jeroen Aerts, Niels Vandamme, Ruth Seurinck, Kevin Verstaen, Gert Hulselmans, Silvie Van den Hoecke, Isabelle Scheyltjens, Kiavash Movahedi, Hans Wils, Joke Reumers, Jeroen Van Houdt, Stein Aerts, Yvan Saeys

**Affiliations:** 1 VIB Tech Watch, VIB Headquarters, Ghent, Belgium; 2 Data Mining and Modelling for Biomedicine, VIB Center for Inflammation Research, Ghent, Belgium; 3 VIB Center for Brain & Disease Research, Leuven, Belgium; 4 Department of Applied Mathematics, Computer Science and Statistics, Ghent University, Ghent, Belgium; 5 Department of Human Genetics, KU Leuven, Leuven, Belgium; 6 Myeloid Cell Immunology Lab, VIB Center for Inflammation Research, Brussels, Belgium; 7 Laboratory for Molecular and Cellular Therapy, Vrije Universiteit Brussel, Brussels, Belgium; 8 Discovery Sciences, Janssen Research & Development, Pharmaceutical Companies of Johnson & Johnson, Beerse, Belgium

**Keywords:** Hashing, scRNA-seq, MULTI-seq, CITE-seq, Sample multiplexing

## Abstract

**Background:**

Multiplexing of samples in single-cell RNA-seq studies allows a significant reduction of the experimental costs, straightforward identification of doublets, increased cell throughput, and reduction of sample-specific batch effects. Recently published multiplexing techniques using oligo-conjugated antibodies or -lipids allow barcoding sample-specific cells, a process called “hashing.”

**Results:**

Here, we compare the hashing performance of TotalSeq-A and -C antibodies, custom synthesized lipids and MULTI-seq lipid hashes in four cell lines, both for single-cell RNA-seq and single-nucleus RNA-seq. We also compare TotalSeq-B antibodies with CellPlex reagents (10x Genomics) on human PBMCs and TotalSeq-B with different lipids on primary mouse tissues. Hashing efficiency was evaluated using the intrinsic genetic variation of the cell lines and mouse strains. Antibody hashing was further evaluated on clinical samples using PBMCs from healthy and SARS-CoV-2 infected patients, where we demonstrate a more affordable approach for large single-cell sequencing clinical studies, while simultaneously reducing batch effects.

**Conclusions:**

Benchmarking of different hashing strategies and computational pipelines indicates that correct demultiplexing can be achieved with both lipid- and antibody-hashed human cells and nuclei, with MULTISeqDemux as the preferred demultiplexing function and antibody-based hashing as the most efficient protocol on cells. On nuclei datasets, lipid hashing delivers the best results. Lipid hashing also outperforms antibodies on cells isolated from mouse brain. However, antibodies demonstrate better results on tissues like spleen or lung.

**Supplementary Information:**

The online version contains supplementary material available at 10.1186/s13059-022-02628-8.

## Background

Recent advances in single-cell and single-nucleus RNA sequencing (scRNA-seq and snRNA-seq) have had an unprecedented impact on our understanding of heterogenous cell populations [1–6]. Current scRNA-seq experiments make it possible to routinely assay many thousands of cells at once, with recent datasets reporting hundreds of thousands to millions of cells [2, 6, 7]. In standard single-cell workflows, individual samples need to be processed in parallel, which limits the throughput, increases reagent costs and has the potential to introduce batch effects. Recently, several approaches for multiplexing have been described, including the use of pre-existing genetic diversity [8] or by introducing sample-specific barcodes using oligo-labeled antibodies [9], oligo-labeled lipid anchors [10], chemical labeling with oligos [11], or genetic cell labeling [12]. Multiplexing samples by labeling cells or nuclei with sample-specific barcodes before pooling and single-cell compartmentalization, a technique called “hashing,” allows for accurate detection of two (doublets) or more (multiplets) cells originating from different samples but captured in the same compartment, which inevitably occurs in standard single-cell workflows. Therefore, implementing a barcoding multiplexing paradigm allows users to drastically increase the number of cells or nuclei loaded per reaction, which consequently decreases per-cell library preparation cost.

The development of oligo-labeled antibodies directed against cell surface proteins for sample multiplexing, is a direct evolution from the Abseq [13], REAP-seq [14], and CITE-seq [15] protocols. One of the most widely used methods to date for detection of the cell epitome is by using the TotalSeq antibodies from Biolegend in combination with the scRNA-seq technologies from 10x Genomics. There are several types of TotalSeq antibodies to be used for cell labeling, including TotalSeq-A antibodies that contain a poly-A sequence mimicking a natural mRNA. These are designed to work with any sequencing platform that relies on poly-dT oligonucleotides as the mRNA capture method, while TotalSeq-B and TotalSeq-C antibodies contain a capture sequences that are compatible with the 10x Genomics 3’ scRNA-seq (v3 or v3.1) and 5’ scRNA-seq workflows, respectively. For human samples, the pre-mixed TotalSeq hashtag reagents recognize cell surface markers CD298 and β2-microglobulin. The success of using antibodies for hashing depends on the ubiquitous expression of these target antigens, which can be problematic for some samples or species [16, 17], limiting the sample-agnostic, universal application of this method. An elegant way to overcome this limitation is the use of lipid anchors that are antigen independent and insert universally into the cell or nucleus membrane, irrespective of sample type [10]. Both antibody-based and lipid-based methods are simple, straightforward and generally applicable to a wide range of single cell applications and platforms, while genetic cell labeling and chemical labeling with oligonucleotides can be more challenging. It is still unclear which method is most accurate in separating samples based on the inserted hashtags. In terms of labour intensity both hashing methods are comparable.

In this study, we compared antibody-based and lipid-based sample barcoding methods by multiplexing four distinct human cancer cell lines. By exploiting the intrinsic genetic variations of these cell lines, demultiplexing by genetic diversity serves as a “ground truth” and allows determining the hashing accuracy of each method.

Single-cell suspensions are most commonly prepared from fresh tissues, which is a major roadblock for analysing clinical samples, archived materials and tissues such as the brain, for which cells cannot be readily extracted [5, 18, 19]. To overcome these limitations, single-nuclei can be extracted and analysed similarly to a standard single-cell workflow [20]. Therefore, we included nuclei samples in our comparison of hashing methods. We also evaluated antibody and lipid hashing on different mouse tissues. Finally, we evaluated hashing accuracy of human PBMCs using TotalSeq-A, -B antibodies and CellPlex reagents, which can be very relevant for single-cell sequencing in clinical studies.

## Results

### Cell hashing using antibodies and lipids accurately demultiplexes majority of cells

**Fig. 1.**
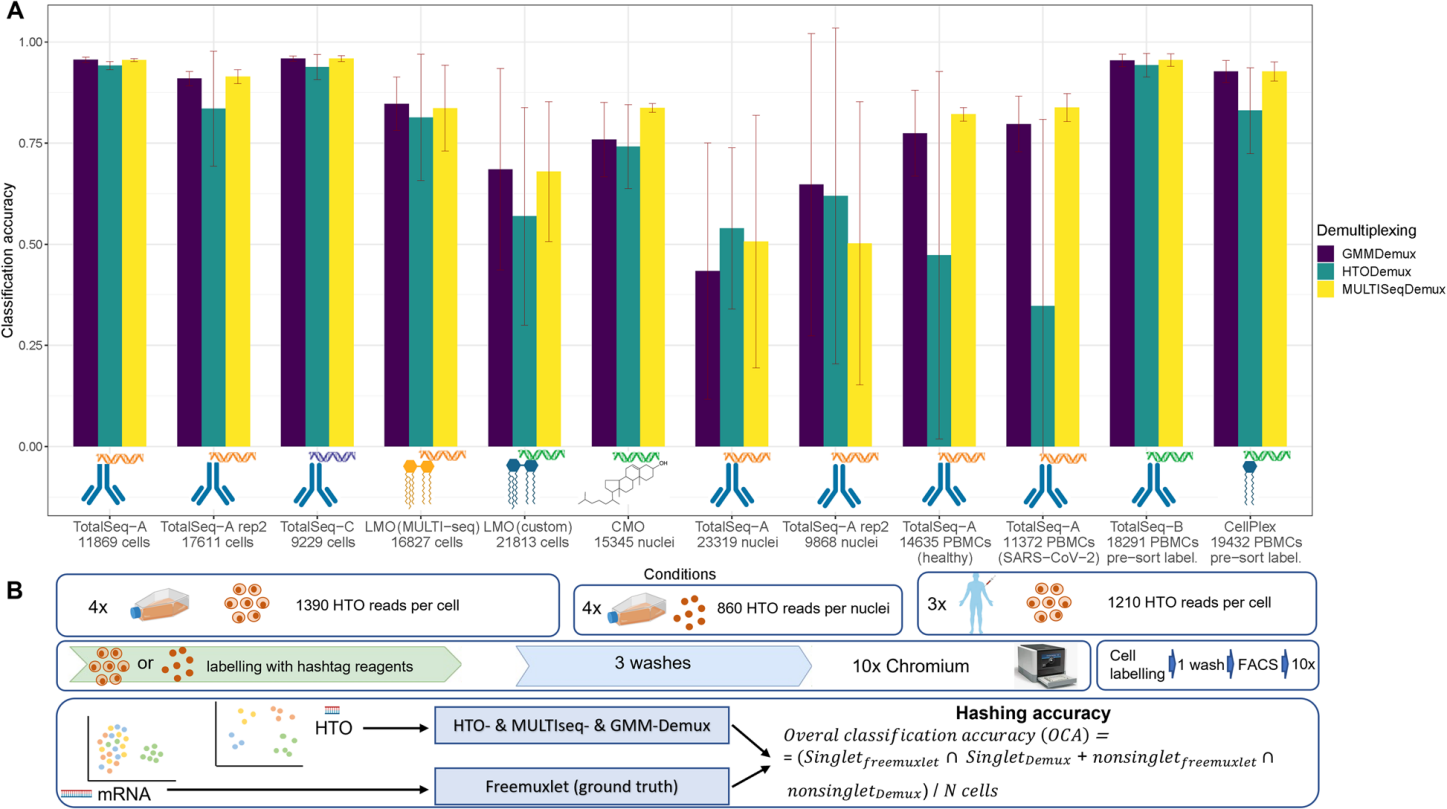
Classification accuracy on human cells and nuclei with an overview of the experimental setup. **A** Overall classification accuracy (OCA) for all tested conditions and demultiplexed functions was calculated using freemuxlet demultiplexing as ground truth. SD represents variations of OCA across 4 cell lines or 3 individuals for PBMCs. **B** Four cancer cell lines or PBMCs were used to extract cells or nuclei to process further with the different labeling methods as indicated on the scheme. After pooling, the samples were run on a 10x Genomics Chromium platform and libraries were sequenced. LMO: lipid-modified oligonucleotides; CMO: cholesterol-modified oligonucleotides. “Pre-sort labeling”—labeling with hashing reagents followed by one wash and live/dead sorting with subsequent loading of the cells on a 10x Genomics chip. Also for these 2 PBMC samples cDNA libraries were generated using dual sample indexing (for all other samples single-sample indexing used)

We first compared antibody-based and lipid-based hashing techniques by pooling four hashed cancer cell lines (MCF7, PC3, DU145, and MDA-MB-231) into a single 10x Chromium run followed by a single analysis pipeline (Fig. 1) for each type of hashing. The hashing accuracy or Overall Classification Accuracy (OCA) was calculated as the overlap between cell line annotation using Seurat (MULTISeqDemux and HTODemux functions) or GMM-Demux [21], and freemuxlet as a reference annotation as follows: the OCA equals the number of all matching singlets (e.g., a given cell *i* annotated as one cell line/strain by both Seurat and freemuxlet), plus a number of matching non-singlets (mostly multiplets),divided by the number of all cells (Fig. 1).

Importantly, the tested demultiplexing functions from Seurat use antibody- or lipid-derived hashtag oligo (HTO) expression data for cell line annotation, while freemuxlet uses transcriptome data and a Bayes Factors approach to evaluate the likelihood of a hashed droplet being a doublet and estimation of its cell line origin. Freemuxlet exploits a similar algorithm as demuxlet [8], but does not require externally genotyped data (popscle package).

**Fig. 2:**
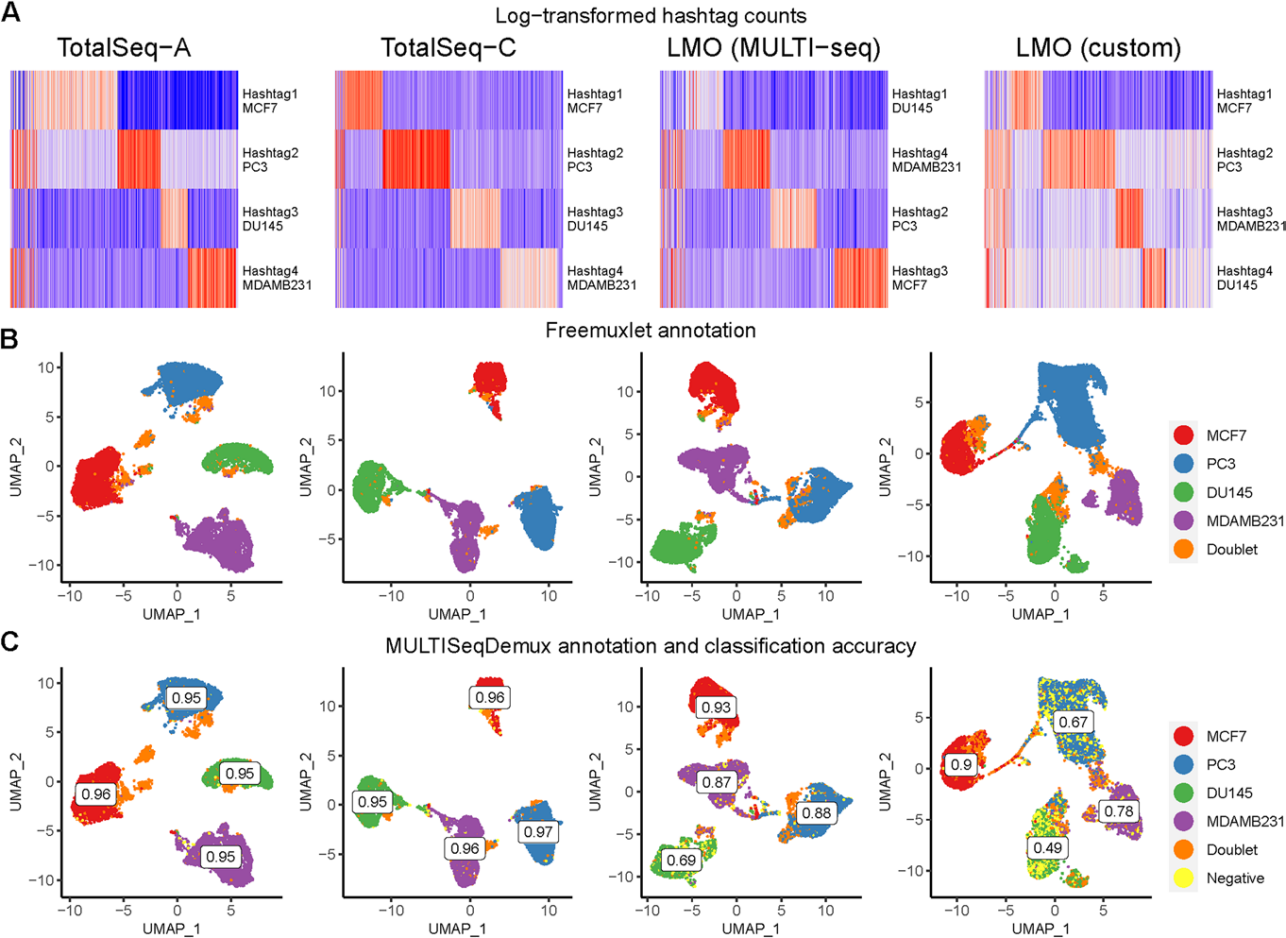
Antibody hashing on cells demonstrates better accuracy than lipid hashing. Each column represents a separate hashing method. **A** Hashtag-derived oligo (HTO) matrices were generated using CellRanger, followed by log-transformation and visualised on heatmaps. **B** Cell annotation (4 cell lines) was performed using freemuxlet (gene expression) and visualized on the gene expression UMAP plots. **C** MULTISeqDemux-annotated cells (HTO signal) were matched with the freemuxlet-annotated cells. Classification accuracy of every hashing method reported for each cell line

One of the first characteristics evaluated for all types of hashing techniques in our study was the level of detection of each HTO. When comparing the unnormalized, log-transformed HTO counts of each antibody- or lipid-derived hashtag oligo on a heatmap, we observed a distinct barcode for each of the four cell line clusters. Each cell cluster expresses an intrinsic gene marker relevant to each cell line (Additional file 1: Fig. S1). These findings are corroborated when each cluster is identified based on inherent genetic variation using freemuxlet (Fig. 2B), clearly separating the four cell lines and doublets. We also evaluated sample multiplexing on the same cells using 5’ scRNA-seq chemistry and compatible TotalSeq-C hashing antibodies (Fig. 2, 2nd column). Cells demultiplexed using MULTISeqDemux had a very high concordance (OCA 0.96 for TotalSeq-A and TotalSeq-C hashing) with freemuxlet-based annotation (Figs. 1 and 2C). The custom lipid (LMO) and MULTI-Seq lipid hashing techniques demonstrated 0.68 and 0.84 classification accuracy, respectively (Figs. 1 and 2C). The MULTISeqDemux function annotated 1.1%, 1.8%, 21%, and 7% of cells in TotalSeq-A, TotalSeq-C, custom lipid (LMO), and MULTI-Seq lipid experiments respectively, as “Negatives” (Fig. 2C), which have a background expression for each hashtag according to the algorithm of the MULTISeqDemux function [10]. We observed ~2 times less unique molecular identifiers (UMIs) and ~3 times less genes per cell in “Negatives” compared to singlets in the case of antibody hashing (no statistical tests performed) (Additional file 1: Fig. S2). Although a number of hashtag reads per cell is normalized across all the compared conditions here, it is noticeable that lower classification accuracy when using lipids compared to TotalSeq antibodies coincides with higher number of cells in the lipid hashing samples (Fig. 1). Nevertheless, the repeated TotalSeq-A experiment with an increased number of cells (17611) still demonstrated better classification accuracy (0.91) even when compared to the MULTI-Seq lipid hashing (0.84, 16,827 cells) (Fig. 1). Remarkably, the number of “Negatives” was relatively higher in DU145 cells hashed with both types of lipid hashes (custom and MULTI-seq) (Fig. 2C), which is in line with the relatively low hashtag level of detection in this cell line (Fig. 2A). This observation might point to a potentially lower affinity of the tested lipids with DU145 cells. When we compared doublet annotation by freemuxlet and MULTISeqDemux, we noticed that 2.4%, 6.3%, 37.9%, and 19.1% of freemuxlet-annotated doublets in TotalSeq-A, TotalSeq-C, custom lipid (LMO), and MULTI-Seq lipid experiments respectively, were recognized as singlets by the MULTISeqDemux function (Additional file 1: Table S1). We observed some variation across different hashing techniques in doublet annotation by different doublet detection tools including also scrublet and doubletFinder (Additional file 1: Fig. S3, S4, S5).

When comparing the correct assignment of cells by the MULTISeqDemux function with their attributed genotypes, the percentage of hashtag swapping (fraction of each hashtag found among singlets in non-labeled cell lines) varied considerably between technologies with the lowest average value of ~ 0,1% mislabeled cells in both TotalSeq-A and TotalSeq-C hashing experiments, 0.89% mislabeled cells in the MULTI-Seq LMO hashing, and 2.68% in the custom LMO hashing experiment (Additional file 1: Table S2).

Regarding the performance of the demultiplexing functions from Seurat, we noticed that the MULTISeqDemux function (autoTresh =T) overall correctly deconvolves more cells than the HTODemux function (with default parameters) or GMM-Demux (Fig. 1).

Additional to the comparison of different hashing techniques on cells, we also estimated the level of expression of CD298 and β2-microglobulin in different human cells (mainly cancer cell lines) using flow cytometry. Both antigens are targeted by human TotalSeq -A and -C hashing antibodies, and for their detection, we used flow cytometry antibodies with the same clones. All the tested 17 human cell types including PBMCs, HEK293A, and THP-1 cells express both antigens, potentially enabling antibody-based hashing on these cells (Additional file 1: Fig. S7). To conclude, the Totalseq -A and -C hashing antibodies can provide good hashing capabilities for a range of human sample types.

### Lipid (CMO)-based hashing outperforms antibody hashing on nuclei

**Fig. 3:**
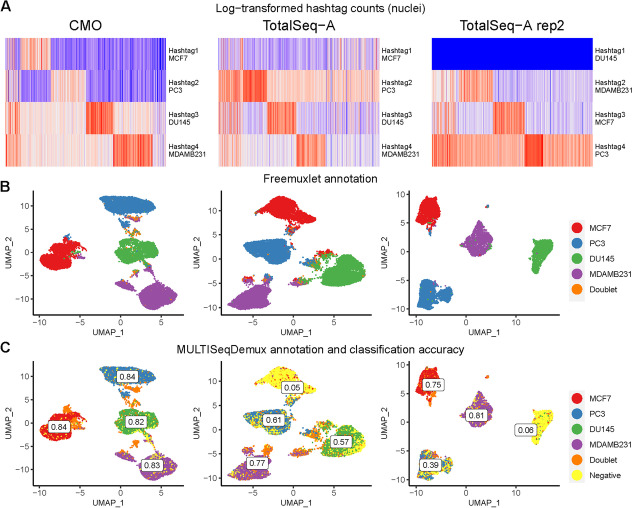
Lipid hashing (CMO) outperforms antibody hashing on nuclei. Each column represents a separate hashing method or a replicate. **A** Hashtag-derived oligo (HTO) matrices were generated using CellRanger, followed by log-transformation and visualised on heatmaps. **B** Nuclei annotation (4 cell lines) was performed using freemuxlet (gene expression) and visualized on the gene expression UMAP plots. **C** MULTISeqDemux-annotated nuclei (HTO signal) were matched with the freemuxlet-annotated nuclei. Classification accuracy of every hashing method reported for each cell line

For each of the four cell lines, we extracted the nuclei to compare the antibody-based and lipid-based hashing efficiency. Nuclei hashing using lipids (cholesterol-modified oligos or CMO) and TotalSeq-A antibodies demonstrated overall lower signal-to-noise ratio of hashtag expression compared to TotalSeq antibody cell hashing (Fig. 2A vs Fig. 3A). Nevertheless, the nuclei hashed with cholesterol oligos followed by demultiplexing using MULTISeqDemux demonstrated 0.84 OCA (concordance with the reference annotation by freemuxlet) or 0.74 when using the HTODemux function (Fig. 1). TotalSeq-A-based nuclei hashing was less accurate (0.51 for MULTISeqDemux and 0.54 for HTODemux function), mainly due to a high number of nuclei recognized as “Negatives” (cells with a background signal for each hashtag) by the demultiplexing functions used for this benchmarking study (Fig. 3C). It was especially prominent in the MCF7 cell cluster (Fig. 3C, 2nd column). However, it was possible to improve the hashing efficiency by adjusting parameters of the demultiplexing functions. For example, changing the “positive.quantile” parameter in the HTODemux function from a default value of 0.99 to 0.9, increased the classification accuracy of the TotalSeq antibody nuclei dataset by more than 10% (from 0.54 to 0.65) (Additional file 1: Fig. 6). We also observed that a large number of negatives in the MCF7 cluster (Fig. 3C) can be explained by an issue with the “hashtag 1” antibodies, since when other cells (DU145) were stained with antibodies from the same vial in a repeated experiment, many DU145 cells were also recognized as “Negatives” by Seurat (Fig. 3B, C, 3rd column). Importantly, CMO nuclei hashing still outperformed the TotalSeq A nuclei hashing (0.84 % vs 0.65 %), when hashtag 1 (DU145) was excluded from the hashing efficiency calculation and compared to CMO nuclei hashing on three cell lines.

Interestingly, the tested hashing techniques on nuclei were characterised by an opposite pattern compared to cell hashing with respect to the background expression for each hashtag (reflected in the number of detected “Negatives”). Accordingly, the MULTISeqDemux function annotated more droplets (33%) as “Negatives” in antibody nuclei hashing compared to the CMO nuclei hashing experiment (8.4% droplets detected as negatives) (Fig. 3C, 2nd column vs 3C, 1st column). This contrasts with the hashing of cells, where antibody hashing delivered less “Negatives” compared to the lipid hashing (Fig. 2C vs Fig. 3C).

The mislabeling hashtag rate for the nuclei samples varied from 0.89% of swapped labels among singlets for CMO technique to ~ 4% for TotalSeq-A nuclei hashing (Additional file 1: Table S2).

We also wanted to compare gene expression-related metrics in samples from the hashing experiments of this study and other non-hashed samples on the single cell line normalized to the same sequencing depth. We did not observe major differences in the number of genes and UMIs per nucleus when comparing MCF7 nuclei from hashing (antibody and lipid) and non-hashed experiments (Additional file 1: Fig. S9). The same conclusion was valid for all the tested hashing techniques on MCF7 cells (Additional file 1: Fig. S10).

In general, opposite to the cell hashing, we observed better hashing results on nuclei when using lipid-based hashing (CMO) compared to the antibody (TotalSeq-A) nuclei hashing.

### Antibody hashing can be successfully applied on clinical PBMC samples

**Fig. 4:**
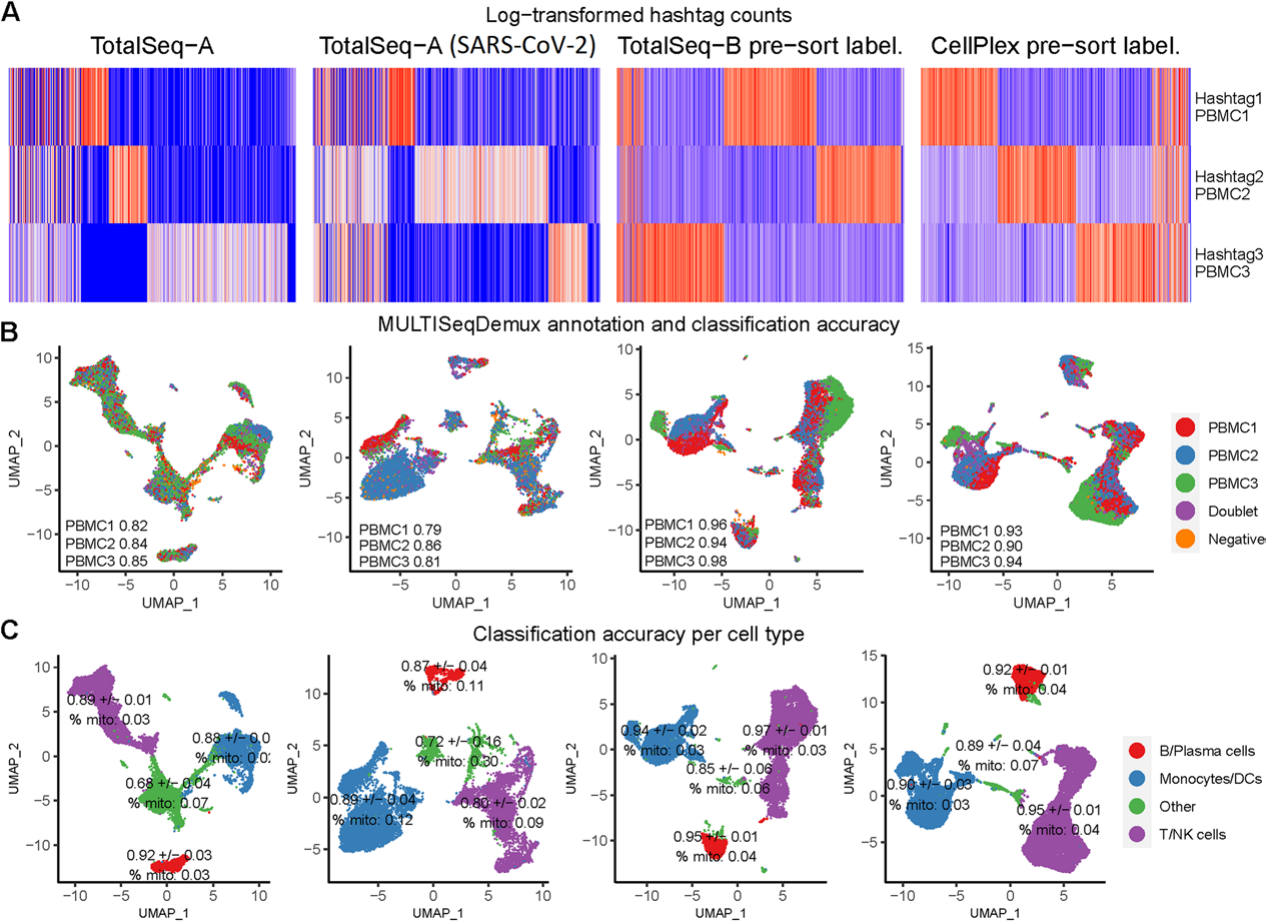
Hashing of human PBMCs including SARS-CoV-2 clinical samples. Each column represents a separate hashing method or a condition (e.g., SARS-CoV-2, 2nd column). “Pre-sort labeling”—labeling with hashing reagents followed by one wash and live/dead sorting with subsequent loading of the cells on a 10x Genomics chip. Other samples undergone “classical” hashing -labeling followed by 3 washes and loading on a chip. **A** Hashtag-derived oligo (HTO) matrices were generated using CellRanger, followed by log-transformation and visualised on heatmaps. **B** MULTISeqDemux-annotated cells (HTO signal) were matched with the freemuxlet-annotated cells (gene data from 3 individuals) and visualized on the gene expression UMAP plots. Classification accuracy of every hashing method reported for each individual. **C** Annotation of major cell types performed using gene expression data and clustering. Classification accuracy of every hashing method reported for each cell type with SD values representing variation across 3 individuals. % mito—percentage of mitochondrial genes for each cell type

In the context of the COVID-19 virus pandemic, we also tested TotalSeq-A antibody hashing on PBMCs from healthy individuals and SARS-CoV-2 patients in the frame of a COVID-19 clinical study (NCT04326920). All samples additionally contained a large 277 antibody CITE-seq (TotalSeq-A antibodies) panel, thus making this PBMC hashing evaluation even more relevant for other multiomics scRNA-seq experiments. The overall classification accuracy (OCA) for the pool of PBMCs from 3 healthy patients according to MULTISeqDemux (autoTresh = T) function was 0.82. For the pool of 3 SARS-CoV-2 patients, it was 0.84. (Fig. 1). Cell clustering analysis demonstrated a presence of all major cell types (T/NK cells, B cells, DCs/monocytes etc.) across 3 individuals in both pools, with no big variation of hashing accuracy across the main cell types (Fig. 4C, 1st and 2nd columns). Remarkably, cell clusters grouped as “Other”, that contain other cell types including potentially dying T-, B-, NK- cells or monocytes (indicated by a higher percentage of mitochondrial genes), had lower hashing accuracy (Fig. 4C).

The MULTISeqDemux function annotated 5.2% and 6.3% cells as “Negatives” in antibody hashing of healthy and diseased samples, respectively (Fig. 4B). According to freemuxlet, the hashtag mislabeling rate was 4.76% of swapped singlet labels for healthy sample and 1.53% for SARS-CoV-2 sample (Additional file 1: Table S2).

Next, we also evaluated the TotalSeq-B and CellPlex (a multiplexing solution from 10x Genomics) hashing on human PBMCs. Since these 2 samples were not part of the abovementioned COVID-19 study, we opted here for another labeling protocol (so called “pre-sort labeling”); labeling with antibodies or lipids followed by only one wash and subsequent live/dead cell sorting (FACS). This resulted in a very high OCA: 0.96 for TotalSeq-B antibodies and 0.93 for CellPlex hashes (Fig. 4, 3rd and 4th columns). It is also important to mention that the CellPlex cDNA libraries are only compatible with the 10x Genomics dual sample indexing (i5 and i7). Therefore, this type of sample indexing was used for the TotalSeq-B and CellPlex hashing here, in contrast to all other cDNA libraries in our study where single sample 10x Genomics indexing (i7) was used.

### Antibody and lipid hashing of primary mouse tissues

scRNA-seq experiments on primary tissues are usually less straightforward when compared to cell lines or PBMCs due to the preceding tissue dissociation steps. In our study we evaluated antibody and lipid hashing of several mouse tissues derived from different mice strains using multiple dissociation protocols and labeling strategies.

First, we evaluated TotalSeq-B antibodies, custom and MULTISeq LMO hashing on the brain (adapted Miltenyi Biotech Neural Tissue Dissociation kit), spleen, lung, and skin cells where we applied “pre-sort labeling.” We observed that the OCA for brain cells was higher when using lipid hashing: 0.87 when using custom LMOs, 0.85 with MULTISeq lipids, and only 0.38 with antibodies. The antibody hashing resulted in many “Negatives” assigned to mostly non-microglia/macrophage cells (*C1qa*-negative cells) (data not shown).

**Fig. 5:**
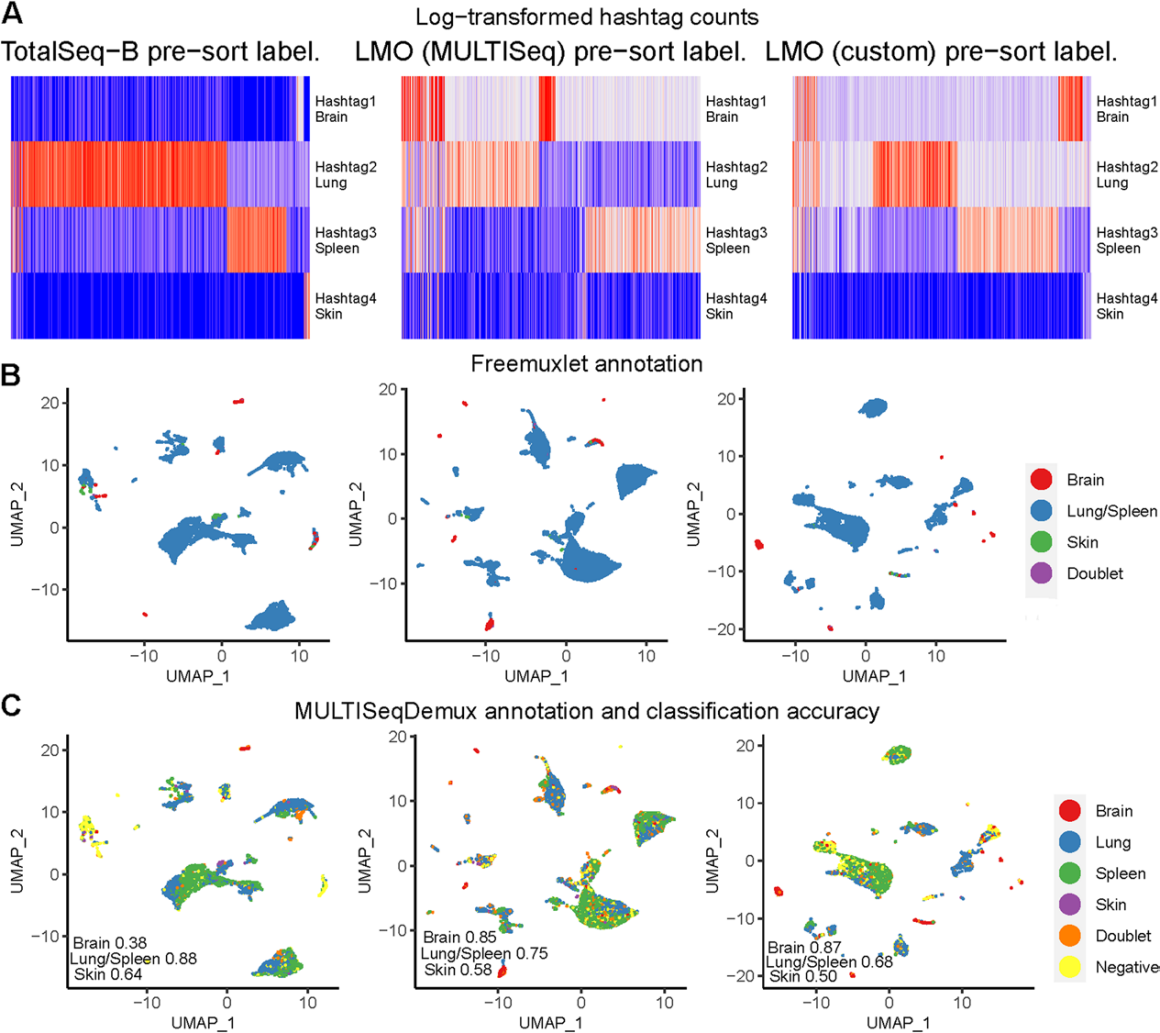
Antibody and lipid hashing of mice brain, spleen, lung, and skin cells. Each column represents a separate hashing method. “Pre-sort labeling”—labeling with hashing reagents followed by one wash and live/dead sorting with subsequent loading of the cells on a 10x Genomics chip. **A** Hashtag-derived oligo (HTO) matrices were generated using CellRanger, followed by log-transformation and visualised on heatmaps. **B** Cell annotation (4 mice strains) was performed using freemuxlet (gene expression) and visualized on the gene expression UMAP plots. **C** MULTISeqDemux-annotated cells, (HTO signal) were matched with the freemuxlet-annotated cells. Classification accuracy of every hashing method reported for each tissue

We could isolate only a few skin cells in this experiment which demonstrated 0.64, 0.5 and 0.58 OCA for antibody, custom LMO and MULTISeq lipid hashing, respectively. We also learned from this experiment that freemuxlet is not able to separate cells originating from closely related mice strains: C57BL6J and C57BL6N. Thus, we report here a common OCA for the spleen and lung. Nevertheless, we could see that antibody hashing (0.88 OCA) outperforms lipid hashing (0.68 OCA for custom and 0.75 for MULTISeq) on the lung and spleen cells (Fig. 5B). We then confirmed this result for antibody hashing in another experiment where the spleen and lung cells were isolated from distinct mice strains: 129SVEvs7 and C57BL6J. The spleen hashing resulted in OCA 0.92 and lung antibody hashing - 0.84. Here we also evaluated a rather classical “post-sort labeling” hashing strategy where sorted live cells were labeled with antibodies followed by 2 washes (OCA 0.92 and 0.8 for the spleen and lung, respectively). Unfortunately, the brain cells were contaminated with blood during isolation in this experiment. As a result, the MULTISeqDemux function annotated basically all brain cells as “Negatives” in the “pre-sort labeling” condition (Additional file 1: Fig. S11, 1st column). In the “post-sort labeling” sample, the OCA for brain cells was only 0.30 (Additional file 1: Fig. S11, 2nd column), which is even lower than in the “pre-sort” labeling condition of the previous experiment where it was 0.38 for antibody brain cell hashing (Fig. 5).

**Fig. 6:**
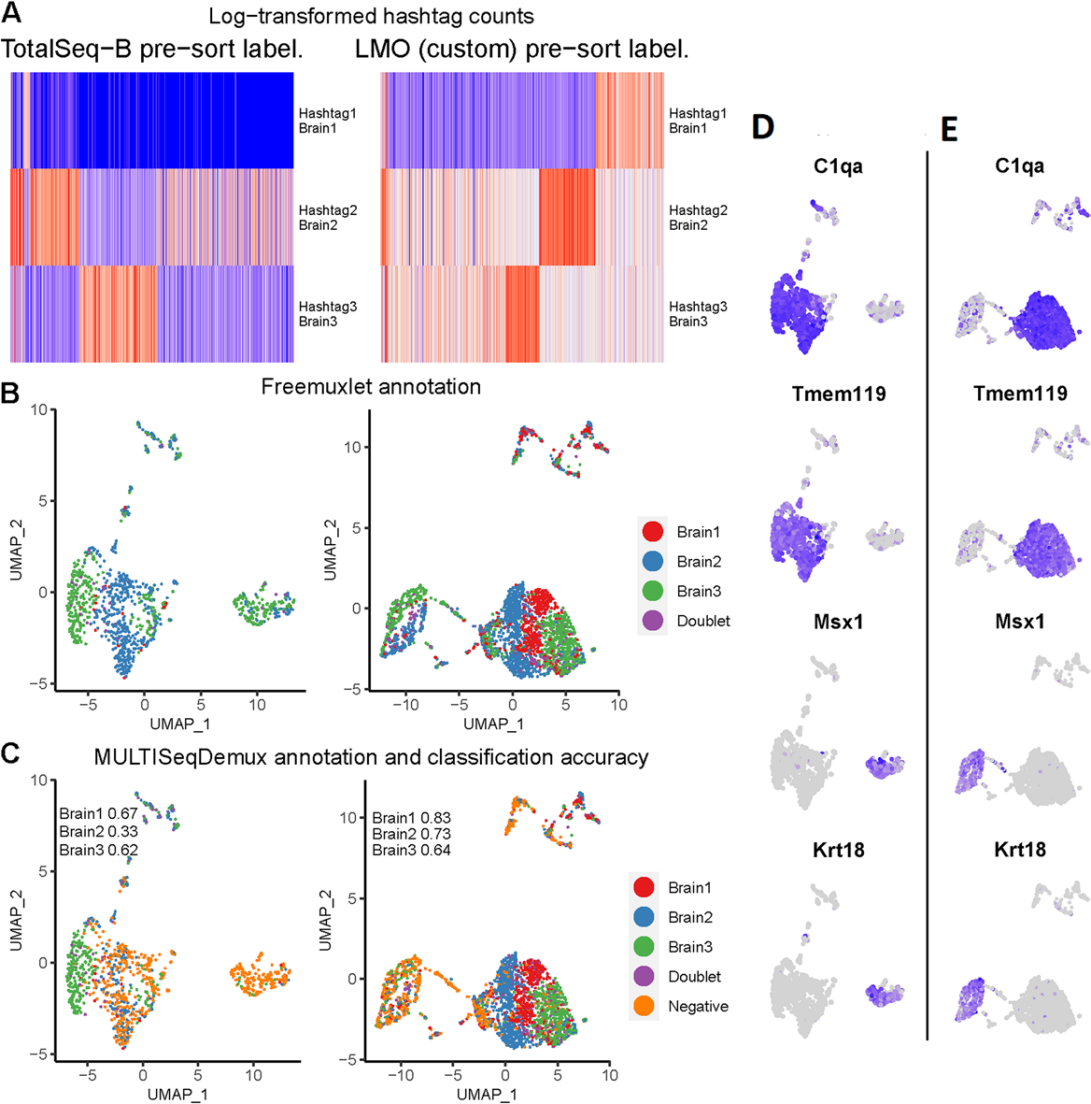
Antibody and lipid hashing of mice brain cells. Each column represents a separate hashing method. “Pre-sort labeling”—labeling with hashing reagents followed by one wash and live/dead sorting with subsequent loading of the cells on a 10x Genomics chip. **A** Hashtag-derived oligo (HTO) matrices were generated using CellRanger, followed by log-transformation and visualised on heatmaps. **B** Cell annotation (3 mice strains) was performed using freemuxlet (gene expression) and visualized on the gene expression UMAP plots. **C** MULTISeqDemux-annotated cells (HTO signal) were matched with the freemuxlet-annotated cells. Classification accuracy of every hashing method reported for each strain (brain cells). **D** Gene-cell matrices were generated using CellRanger, followed by log-transformation of gene UMI counts and cell clustering (gene expression, PCA reduction) using Seurat. The marker gene UMI counts were visualised in blue color on the gene expression UMAP plots for antibody hashing and lipid hashing (**E**)

Next, we decided to evaluate antibody and lipid hashing on brain cells using another isolation protocol which also includes transcardial perfusion to prevent blood contamination [4]. In this case, lipid hashing (custom lipids) also demonstrated better hashing accuracy than antibodies (OCA 0.73 vs 0.48) (Fig. 6). Here, we also applied hashing before live/dead cell sorting (“pre-sort labeling” protocol). Regarding the cells that could not be assigned to any hashtag (“Negatives”), even in the lipid hashing sample, these were the cells mostly belonging to cell clusters that express markers of ependymal cells (*Msx1, Krt181*, etc) [22]. The cells which were mostly successfully demultiplexed when using lipid hashing are microglia/macrophages (*Tmem119/C1qa* positive cells) [4] (Fig. 6D, E).

## Discussion

Correct sample demultiplexing after hashing depends on several factors: the hashing strategy itself (antibodies versus lipids), which subsequently can depend on the cell surface antigen repertoire in case of antibody hashing or biochemical/biophysical properties of membranes in case of lipid hashing. Also, this accuracy depends on the demultiplexing methods that can be subsequently influenced by other parameters (cell number, cells or nuclei, sequencing depth, etc.). In order to accurately determine the efficiency of demultiplexing, we hashed different cell lines that carry intrinsic genetic variations, which serve as a ground truth control. Importantly, for one of the hashing datasets, we compared two reference demultiplexing tools: freemuxlet which was used in this study, and the genotyping data-dependent tool demuxlet [8]. The cell deconvolution of the “TotalSeq-A cells rep1” sample using HTODemux demultiplexing function demonstrated a 0.94 OCA (overall classification accuracy) when compared with freemuxlet, and 0.95 when compared with demuxlet as a reference. Our comparison demonstrates that even without external genotyping data (SNPs), required for demuxlet, it is possible to deconvolve different cells using a similar algorithm (freemuxlet). Although it is possible to demultiplex samples using only freemuxlet when pooling cells from different patients or from distinct mouse strains, the tool fails to separate strains such as C57BL6N and C57BL6J for example. We also noticed that “doublet vs singlet” classification by freemuxlet was not always consistent with the annotation by DoubletFinder [23] or Scrublet, the commonly used tools for computational doublet prediction [24]. For some data sets freemuxlet was underestimating the number of doublets. Additionally, the doublets annotated by HTO- and MULTISeqDemux were also not always aligned with the output of DoubletFinder and Scrublet. These findings are in accordance with the observation of a relatively high disagreement between DoubletFinder, Scrublet, and demuxlet [25]. This can be partially explained by the reliance on different data modalities that can lead to a certain bias in detection of heterotypic or homotypic doublets. For instance, the functions incorporated in Seurat (e.g., HTO-Demux, MULTISeqDemux) predict doublets based on the hashtag signal (in particular combinations of different hashtags), while other tools (e.g., DoubletFinder) use the gene expression data and can detect doublets formed by transcriptionally distinct cells [9, 23]. Thus, it is worthwhile to consult both types of methods (gene expression- and HTO-based) while keeping in mind a theoretical multiplet rate (~0.8% for every 1000 captured cells on a 10x Genomics chip, CG000206 Rev D). Overall, this limitation in correct doublet prediction might diminish demultiplexing accuracy and requires further optimization. The impaired doublet annotation due to increased multiplet rate together with a failure to assign hashtags to a number of cells (“negatives”) might explain the lower hashing efficiency in the samples with higher number of cells loaded/captured on chip. For example, the captured cell number in the repeated TotalSeq-A experiment (rep2) was increased by 48%, while the number of doublets (detected by freemuxlet) and negatives (assigned by MULTISeqDemux) increased by 125% and 325%, respectively. An important parameter “signal-to-noise ratio” [22] was kept in this comparison on the same level by labeling equal number of cells with the same quantity of hashing antibodies.

It is important to mention that our comparison of different hashing strategies was applied to cells from filtered gene-cell matrices as produced by CellRanger v3 without further outlier filtering (e.g., based on mitochondrial gene expression). We could see in our PBMC dataset that in terms of cell calling, this filtered matrix contains almost the same number of cells as a “raw” matrix after filtering out all droplets with less than 200 UMIs (gene expression) and all droplets expressing one gene in less than 3 droplets. We observed that the tested demultiplexing functions from Seurat assigned some cells with relatively low number of genes and UMIs to the group “negatives”. Hence, not surprisingly, the hashing efficiency can be improved by filtering out the cell outliers. For example, correct PBMC annotation (healthy control patients) was improved from 0.84 to 0.89 after filtering out 2660 from 14635 cells using the “scater” Bioconductor package (identifying outliers based on the number of genes and UMIs per cell). On the other hand, a stringent filtering might cause a loss of biologically relevant cell subtypes. Thus, optimization of the sample demultiplexing should be performed in parallel with a deeper biological sample analysis. For evaluation of TotalSeq-B antibodies and CellPlex on PBMCs, we opted for a different protocol where labeled cells were sorted (live/dead) before loading on a chip. This “pre-sort labeling” might have contributed to a higher classification accuracy compared to the classical hashing protocol (labeling without sorting). It is in line with another observation that in PBMC clusters with a higher percentage of mitochondrial genes, hashing accuracy was lower.

We can conclude that the type of hashing strategy must be chosen based on the hashtag antigen expression, since it is known that some cells might not express both antigens targeted by available TotalSeq hashing antibodies. For instance, CD45 and MHC I antigens (targeted by mouse hashing antibodies from BioLegend) are not or lowly expressed in mouse C3 and B16-BL6 melanoma cells (Additional file 1: Fig. S8). However, a relatively lower affinity of certain cells towards different types of lipid hashes can be also observed. Nevertheless, lipid-based hashing might be a preferred strategy for multiplexing of samples with a low or unknown expression of hashing antigens (CD45 and MHC I), provided the sample demultiplexing on a computational level is well optimized. That is what we see in a rather complex tissue such as mouse brain, where lipid hashing outperforms antibody hashing. The effect of tissue dissociation on epitope representation can also contribute to this observation in case of antibody hashing. Nevertheless, if working only with for instance brain macrophages (i.e., microglia), the antibody hashing can still be used. The antibody hashing however seems to be a preferred strategy when working with tissues like the spleen or lung where tissue dissociation is more straightforward.

In the present study on human cancer cell lines, the antibody-based hashing when targeting cells overall performed better than the lipid-based hashing. When labeling nuclei from the same cell lines, cholesterol-based hashes (CMO) demonstrated better hashing efficiency compared to the antibody-based labeling (TotalSeq-A). This result can be partially explained by the potential detrimental effect of the lysis buffer (Nuclei EZ Lysis Buffer) used for nuclei isolation on the nuclei surface proteins. The comparison of TotalSeq -A-based cell hashing with TotalSeq-C cell hashing compatible with 3’ and 5’ gene 10x Genomics sequencing, respectively, demonstrates similarly good hashing efficiency. When comparing TotalSeq-B hashing antibodies with CellPlex (hashing lipids from 10x Genomics) on PBMCs, both methods demonstrate very high hashing accuracy—0.96 for antibodies and 0.93 for CellPlex. Alternatively, other antibody-based hashing methods are also available on the market, e.g., hashing antibodies from BD Biosciences compatible with the BD Rhapsody platform for scRNA-seq experiments.

With respect to analysis algorithms, we observed that the MULTISeqDemux function with autoTresh =T parameter originally tailored for lipid-based hashing [10] performs even better than HTODemux function and only slightly better than the GMM-Demux function on a vast majority of tested data sets (Additional file 1: Fig. S12).

## Conclusions

**Fig. 7:**
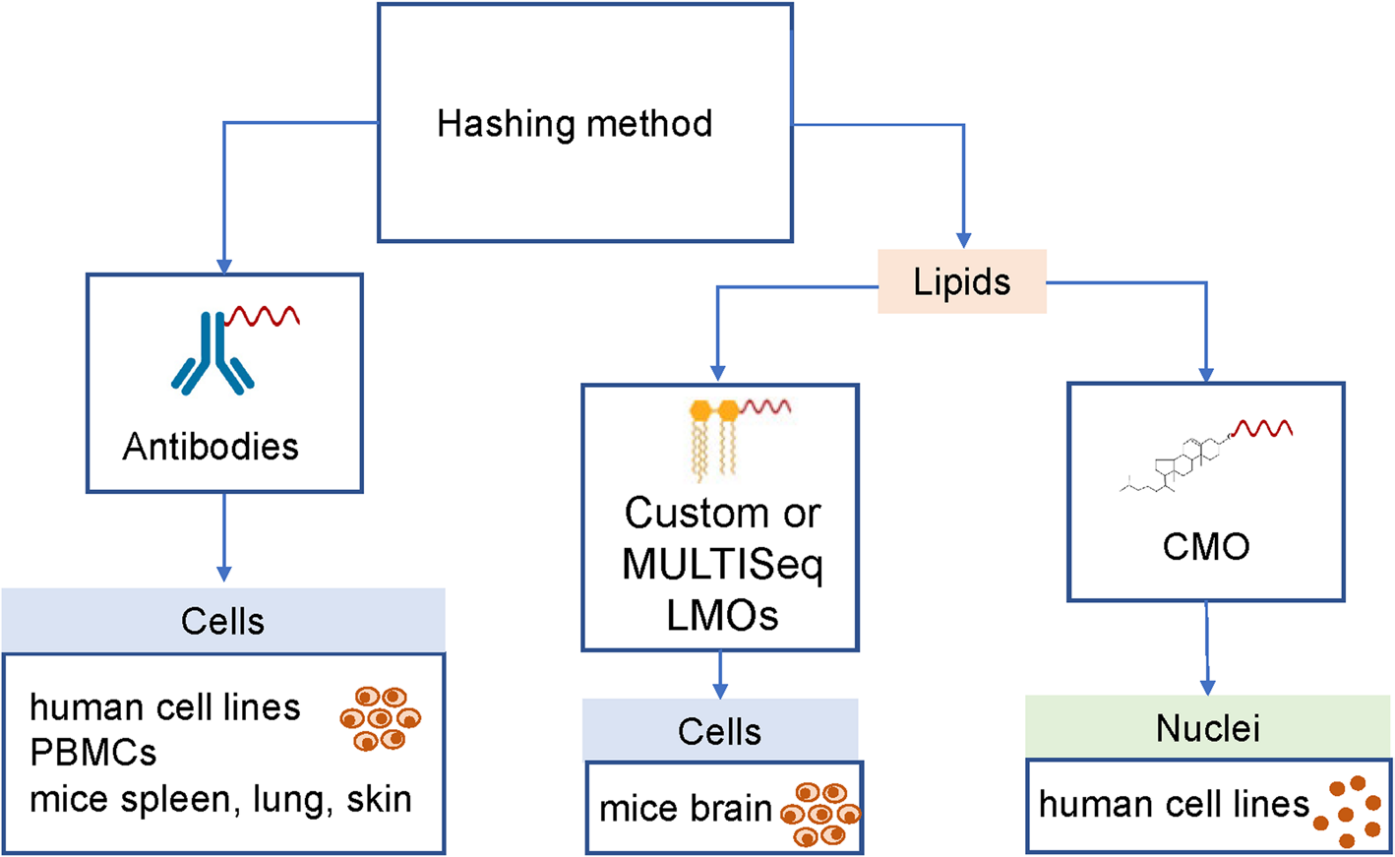
Preferable hashing techniques according to our study. TotalSeq antibody hashing is a preferred method for multiplexing different human cell lines, PBMCs, mice spleen, lung, and skin cells. Lipid-modified oligos are better on mice brain cells. The CMO hashing method yields the best results when nuclei are the input sample. CMO cholesterol-modified oligos

Overall, we can conclude that both lipid- and antibody-based hashing strategies can be successfully applied on human cells, nuclei, and on cells form mouse tissues to reduce costs and batch effects of scRNA-seq experiments. Our comparison indicates that MULTISeqDemux is the preferred demultiplexing function, antibody-based hashing is the most efficient protocol on human cell lines, PBMCs, and lipid hashing delivers the best results on nuclei. On mice tissues, this depends on the tissue, e.g., lipids work better on the brain and antibodies better on the lung and spleen (Fig. 7). We also demonstrate that antibody hashing in combination with large CITE-seq panels can be successfully applied on PBMCs from healthy individuals and SARS-CoV-2 patients.

## Methods

### Cell culture

MCF7, PC3, DU145, and MDA-MB-231 cells were ordered from ATCC via LGC Standards and maintained according to standard procedures in RPMI-1640 (Gibco, #21875034), F-12K (LGCstandards, ATCC 30-2004), EMEM (LGCstandards, ATCC 30-2003), and DMEM: F-12 (LGCstandards, ATCC 30-2006) medium, respectively, supplemented with 10% fetal bovine serum (Gibco, #10082147) and 1% penicillin/streptomycin (100 U/ml and 100 μg/ml, respectively, Gibco, #15140122) at 37°C with 5% CO_2_. RPMI-1640 medium used to culture the MCF7 cells was additionally supplemented with 0.01 mg/ml human recombinant insulin (Sigma Aldrich, #I3536). The cells had undergone regular tests for mycoplasma contamination during the study.

### Single-cell dissociation

To prepare single-cell solutions of the cultured cell lines, the culture medium was removed, and the cells were washed with 1X PBS. Afterwards, the cells were trypsinized (0.05% trypsin-EDTA, Gibco, # 25300054) and pelleted at 200 x *g* for 5min. The cells were resuspended in 2 ml of culture medium, gently mixed with 8 ml of 1X PBS and put on ice. Afterwards, the cells were pelleted at 200 x *g* for 5min on 4°C and resuspended in 1 ml 1X PBS with 0.04% BSA. Cells were passed through a 40-μm cell strainer (Corning, # CLS431750-50EA) and counted with a LUNA FL counter, using 1 μl of acridine orange and propidium iodide dye (F23001, Westburg).

### Single nuclei dissociation

Single nuclei suspensions were prepared using a modified nuclei isolation protocol from 10x Genomics (demonstrated protocol: isolation of nuclei from single-cell suspensions, #CG000124, revD). We replaced the original lysis buffer with Nuclei EZ Prep buffer (Sigma Aldrich, # NUC101-1KT) with a total lysis time of 5 min and proceeded according to the referenced protocol. Loss of cell viability as a proxy for nuclei extraction quality and the number of nuclei were assessed with a LUNA FL counter, using 1 μl of acridine orange and propidium iodide dye (F23001, Westburg).

### Mice primary tissue processing

#### Brain

For the mice (8 weeks old BALB/cJ) brain cell isolation, the Miltenyi Biotech Neural Tissue Dissociation kit (130-092-628) was used without perfusion according to manufacturer’s guideline.

Alternatively the following brain cell isolation protocol was used as described [4]: 8-week-old mice (BALB/cJ, C57BL/6N and DBA/2J) were deeply anesthetized and perfused transcardially with 20 ml of ice-cold phosphate-buffered saline (PBS). Mice were decapitated, and the brains were divided in two hemispheres and collected in 1 mL ice-cold Roswell Park Memorial Institute (RPMI) 1640 medium (Gibco). The brains were cut into small pieces (1–2 mm) after which enzyme mix was added (30 U ml−1 DNAse I (Roche), 10 U ml−1 collagenase type I (Worthington), and 400 U ml−1 collagenase type IV (Worthington) diluted in 1X Hanks’ buffered salt solution (Gibco)). The brains were incubated for 20 min at 37°C and resuspended with a 1-mL pipet every 10 min. The solutions of three hemispheres per condition were pooled and filtered twice over a 100-μm nylon filter and centrifuged. The pellet was resuspended in 5 ml 70% standard isotonic percoll (SIP, GE Healthcare) diluted in 1X Hanks’ buffered salt solution and carefully overlaid with 5 ml of 37% SIP, followed by a 5-ml layer of 30% SIP, forming a three-layered density gradient (centrifuged at 800g, 4°C, 30 min without acceleration/braking). The 70/37% interphase containing immune cells was collected, centrifuged, pooled per condition, and resuspended in fluorescent activated cell sorter (FACS) buffer (2 mM EDTA (Duchefa) and 2% heat-inactivated fetal calf serum (Gibco) dissolved in 1X Hanks’ buffered salt solution). The duration of the entire dissociation process was 4 h.

#### Lung, spleen, and skin

Eight-week-old mice (C57BL6/J and C57BL6/N in the 1st experiment, C57BL6/J and 129SVEvs7 in the 2nd experiment) were used. Single-cell suspensions were prepared by digestion in 100μg/mL Liberase TL (Cat 05401020001, Roche, Germany) and 100μg/mL DNase I (11284932001, Roche, Germany) for 30 min at 37°C before passing through a 100-μm cell strainer. The spleens were rinsed with ice-cold PBS and mashed into small pieces using a plunger of a 10-mL syringe on top of a sterile 100-μm cell strainer mesh in a Petri dish containing 2-ml ice-cold RPMI. Red blood cells in the lung and spleen single-cell suspensions were lysed using ammonium chloride lysis buffer (10 mM KHCO_3_, 155 mM NH4Cl, 0,1 mM EDTA in MilliQ water). Single-cell suspensions were washed with 1x dPBS before incubation with antibodies. The skin cells were isolated as described here [26].

### Antibody hashing

Cell and nuclei labeling were performed according to an adapted BioLegend cell hashing protocol (TotalSeq™-A Antibodies and Cell Hashing with 10x Single Cell 3' Reagent Kit v3 3.1 Protocol, Biolegend). Briefly, cells were then incubated for 10 min with Fc receptor block (PN 422301, TruStain FcX, BioLegend) to block nonspecific antibody binding. Subsequently, cells were incubated with mixtures of barcoded antibodies for 30 min at 4°C. In the TotalSeq-A experiment, MCF7, PC3, DU145, and MDA-MB-231 cells were labeled with 0.1 μg TotalSeq-A0251, -A0252, -A0253, and –A0254, respectively. For TotalSeq-C hashing, MCF7, PC3, DU145, and MDA-MB-231 cells were labeled with 0.1 μg TotalSeq-C0251, -C0252, -C0253, and –C0255, respectively. For both TotalSeq-A and TotalSeq-C experiments, 200 000 cells of each cell line were labeled with the antibodies in 100 μl PBS containing 1% BSA. After labeling, cells were washed 3 times by resuspension in 1.4 ml PBS containing 1% BSA, followed by centrifugation (200g 5 min at 4°C). After the final wash, cells were resuspended in PBS containing 0.04% BSA and equal amounts of labeled MCF7, PC3, DU145, and MDA-MB-231 cells were pooled before loading onto the 10x Chromium Single-Cell B Chip (PN# 2000060) in case of TotalSeq-A hashing or onto the 10x Chromium chip A (PN# 2000019), because of the TotalSeq-C antibody compatibility with 5’ 10x chemistry (Chromium Single Cell Immune Profiling).

Nuclei were labeled with oligo-tagged anti-nucleoporin antibodies (#Mab414, BioLegend) (Sup. Table 1). MCF7, PC3, DU145, and MDA-MB-231 nuclei (840 000 of each line) were labeled with 0.5 μg TotalSeq-A0456, -A0457, -A0458, and –A0459, respectively, in 2% BSA solution with 0.02% Tween-20, 10mM Tris, 146mM NaCl, 1mM CaCl_2_, and 21mM MgCl_2_ [27]. After the last wash, nuclei were resuspended in the same labeling buffer as above with lower concentration of BSA (1%) and pooled prior to loading on chip B from 10x Genomics.

The DNA library preparation was performed according to the manufacturers guidelines: CG000186 Rev A protocol for 5’ 10x Genomics chemistry and CG000185 Rev B protocol for 3’ 10x Genomics (v3 chemistry).

### Lipid hashing

Based on the MULTI-seq work, Lipid Modified Oligonucleotides (LMO) were used for cell hashing, and Cholesterol Modified Oligos (CMOs) were used for nuclei hashing. Labeling of nuclei was done with CMO because of the inefficacy of the LMOs to label nuclei in the presence of BSA. BSA specifically sequesters LMO and quenches LMO labeling [10].

In this work, we have evaluated and compared the performance of commercially available oligos (custom LMOs purchased from Integrated DNA Technologies) with the LMOs used in the MULTI-seq work [10]. Due to the commercial unavailability, LMOs with lower carbon chain length (C18; Stearyl modification) were used instead of the lignoceric acid modified oligonucleotides (C22), described in the MULTI-seq manuscript.

The MULTI-seq lipid anchor and co-anchor were a kind gift from Christopher S. McGinnis at UCSF. The labeling of MCF7, PC3, DU145, and MDA-MB-231 cells was performed as previously described [10]. Briefly, for each cell line, 500k dissociated cells were washed and resuspended in PBS and incubated with 200 nM 1:1 molar ratio of the anchor and unique barcode oligonucleotides for 5 min on ice. Afterwards, each sample was incubated with 180nM of co-anchor in PBS on ice for 5min, followed by addition of 1% BSA in PBS to reduce off-target labeling when pooling. Cells were washed two times in 1% BSA in PBS after which all samples were pooled, filtered through a 40-μm cell strainer (Corning, # CLS431750-50EA), counted and loaded onto a single reaction of a 10x Chromium chip B.

The anchor and co-anchor of the commercial LMOs are available through Integrated DNA technologies. Also, the anchor and co-anchor of the CMOs were conjugated to cholesterol at the 3′ or 5′ ends via a triethylene glycol (TEG) linker and are also commercially available from Integrated DNA Technologies (Additional file 1: Table S4). The labeling workflow for the commercially available LMOs and CMOs are identical to the MULTI-seq protocol [10].

For the mice samples, tissues from different strains were dissociated and 0.4–0.6 M cells were labeled with antibodies (0.5 μg) or lipids (200 nM) as described above. Then cells were washed once (pre-sort labeling protocol) and live cells sorted (BD Aria II, 85 ul nozzle, DAPI staining) for the subsequent cell pooling and 10x Genomics runs. Alternatively, cells were labeled (same amount of antibodies or lipids as above) after sorting (0.3–0.4M cells) and washed 2–3 times before pooling and loading on 10x chips (post-sort labeling).

### Sequencing

The generated libraries were pooled targeting 85% mRNA and 15% hashtag oligo (HTO) and paired-end sequenced on individual lanes on a HiSeq4000 or NovaSeq 6000 (Illumina) instruments, using the following read lengths: 28 bp Read1, 8 bp I7 Index, and 91 bp Read2.

### PBMC sample preparation and single-cell sequencing

PBMCs from 3 healthy control individuals and 3 SARS-CoV-2 patients (in a framework of the clinical study NCT04326920) were isolated as follows. Whole blood separation was performed by bringing whole blood, diluted with PBS 7.2 (ThermoFisher Scientific, # 20012027), in a Leucosep™ tube, (Greiner Bio-One, # 227290), prefilled with 15 mL Lymphoprep™ (Stemcell technologies, # 07851), and followed by a centrifugation step of 30 min at 400g (acceleration 5, brake 3). After isolation, the PBMCs were washed twice with PBS 7.2 and centrifuged at 350g during 10 min at 4°C. Isolated PBMCs were counted, cryopreserved in 1mL FCS/10%DMSO, and stored in liquid nitrogen. After the thawing as described in the PBMC sample preparation protocol from 10x Genomics (CG00039, Rev C), cells were labeled according to the adapted CITE-seq protocol [15]. In brief, 1 x 10^6 PBMCs were spun down and resuspended in 25 μl CITEseq antibody cocktail (TotalSeq™-A Antibodies, BioLegend) containing among others one hashing antibody per each sample (0.025μg). After a 30-min incubation on ice, cells were washed 3 times, pooled together (3 healthy donors resulting in sample one and 3 SARS-CoV-2 patients resulting in sample two), and resuspended in PBS+0.04% BSA at a final concentration of 1000 cells/μl. Cells were counted using automated counter LUNA FL^TM^ (Logos Biosystems). Cellular suspensions (target recovery of 10000 cells) were loaded on a 10x Genomics NextGEM Single-Cell Instrument, chip G (# 2000177) to generate single-cell Gel Bead-in-EMulsion (GEMs). Single-cell RNA-Seq libraries were prepared using GemCode Single-Cell V3.1 (NextGEM) 3′Gel Bead and Library Kit (10x Genomics) according to the manufacturer’s instructions. Sequencing libraries were sequenced with Illumina NovaSeq S4 flow cell with custom sequencing metrics (single-indexed sequencing run, 28/8/0/98 cycles for R1/i7/i5/R2).

For the CellPlex vs TotalSeq-B evaluation, commercial frozen PBMCs from 3 different healthy donors (SER-PBMC-F, tebu-bio) were used according to the 10x Genomics PBMC sample preparation protocol CG00039 Rev C. 0.5 x 10^6 PBMCs for CellPlex were labeled according to the 10x Genomics CG00391 Rev A protocol. The same number of PBMCs were incubated for 10 min with Fc receptor block (PN 422301, TruStain FcX, BioLegend) and then incubated with 0.2 μg of TotalSeq-B0258 or -B0259, or -B0260 for 30 min at 4°C. The cells were washed once and live-sorted on BD Aria II instrument with 85 μm nozzle (DAPI staining). Afterwards, cells were collected and processed according to 10x Genomics CG000390 Rev B protocol. Generated dual index cDNA libraries were sequenced with 10x Genomics dual indexing metrics: 28/10/10/90 cycles for R1/i7/i5/R2 on NovaSeq instrument.

### Single-cell data processing

#### Mapping

The raw sequencing data were demultiplexed and further converted into a single-cell level gene counts matrix with Cell Ranger 3.1.0 with default parameters (https://github.com/10xGenomics/cellranger). Only the number of expected cells was adapted in accordance with the number of targeted (captured) cells in each experiment. The mRNA reads were mapped to the human reference genome (assembly hg38 build 95) with the reads allowed to map to both exonic and intronic regions. For every type of hashing strategy, a specific Feature Reference File was generated and used as input for Cell Ranger. Filtered count matrices were used for downstream analysis. CellPlex and TotalSeq-B PBMC samples from dual index libraries were mapped with cellranger version 6.1.1. Mouse samples were mapped to GRCm38 genome.

#### Freemuxlet-based demultiplexing

The deconvolution of the four cell lines was determined by a genotype-free tool freemuxlet which is an extension of demuxlet [8]. Both tools are from the popscle (commit 7b141e3) software available at https://github.com/statgen/popscle/. To reduce computation time, the input bam file was sorted to contain only reads that (i) overlap with the SNPs in the VCF file and (ii) have a cell barcode listed in the cell barcode list. Code is available at https://github.com/aertslab/popscle_helper_tools. The filtered bam file was further used with the popscle dsc-pileup tool to pileup the reads and corresponding base quality for each overlapping SNPs and each barcode. The reference vcf file was assembled from the 1000 Genomes Project GRCh38 genetic variants data [28] adapted to the index used for mapping and the variant allele frequency > 0.9 and <0.1*.* were discarded. Next, freemuxlet with default parameters was used to determine sample identity and identify doublets. For the mouse samples VCF file from the “Mouse Genomes Project” was used (https://www.sanger.ac.uk/data/mouse-genomes-project/).

#### Sample demultiplexing and clustering

To assign hashing tags for each cell, two strategies implemented in Seurat 3.1.4 package [29, 30] in R version 3.6.0 were evaluated on the filtered feature-barcode matrix generated by CellRanger: (i) HTODemux function with default parameters and (ii) incorporated into Seurat MULTIseqDemux function [10] with argument autoThresh = TRUE. Subsequent clustering and visualization of the data sets was performed in R using Seurat version 3.1.4 functions as described in the “Demultiplexing with hashtag oligos (HTOs)” vignette available at (https://satijalab.org/seurat/v3.1/hashing_vignette.html). The hashing accuracy was calculated as the overlap between cells annotated using MULTISeqDemux, HTODemux functions, or GMM-Demux (https://github.com/CHPGenetics/GMM-Demux) and freemuxlet as a ground truth.

#### Doublet identification

Doublet cell detection was performed using DoubletFinder (v2.0.1) [23] with the 10% as expected doublet rate and the following parameters: PCs=1:25, pN=0.25, pK= the highest value on BCmvn plot (find.pK function); Scrublet (v 0.1.3) and freemuxlet with default parameters, and Seurat (MULTISeqDemux (autoTresh=T) and HTODemux (default parameters) functions). The functions incorporated in Seurat predict doublets based on hashtag signal, while other tools use transcriptome data for this.

## Supplementary Information

**Additional file 1.** Combination of all supplementary figures (Fig S1 –S12) and tables (table S1-S4).

**Additional file 2.** Reviewer History.

## Data Availability

The datasets (raw and processed) supporting the conclusions of this article are available in the European Nucleotide Archive (ENA; https://www.ebi.ac.uk/arrayexpress/experiments/) repository, under accession numbers E-MTAB-9964 [31] and E-MTAB-11401 [32]. The SARS-CoV-2 PBMC datasets used in this work currently are not publicly available due to an ongoing clinical study but are available from the corresponding author on reasonable request.
