# Supplementary material for: Comparative analysis of antibody- and lipid-based multiplexing methods for single-cell RNA-seq
Source: Genome Biol. 2022 Feb 16;23:55. doi: 10.1186/s13059-022-02628-8 (PMC8851857; doi:10.1186/s13059-022-02628-8)
Supplement: Supplementary file 1 — Additional file 1. Combination of all supplementary figures (Fig S1 –S12) and tables (table S1-S4). [file 13059_2022_2628_MOESM1_ESM.pdf]

SUPPLEMENTAL DATA

Comparative analysis of antibody- and lipid-based multiplexing methods for single-cell RNA-seq

Viacheslav Mylka[1,4], Irina Matetovici[1,2], Suresh Poovathingal[2,], Jeroen Aerts[1,2], Niels Vandamme[4,5], Ruth Seurinck[4,5], Kevin Verstaen[4,5], Gert Hulselmans[2,3], Silvie Van Den Hoecke[1], Isabelle Scheyltjens[7,8,],Kiavash Movahedi[7,8], Hans Wils[6], Joke Reumers[6], Jeroen Van Houdt[6], Stein Aerts[2,3,+], Yvan Saeys[4,5,+]

[+] These authors contributed equally

[1] VIB Tech Watch, VIB Headquarters, Ghent, Belgium.
[2] VIB Center for Brain & Disease Research, Leuven, Belgium.
[3] Department of Human Genetics, KU Leuven, Leuven, Belgium.
[4] Data mining and Modelling for Biomedicine, VIB Center for Inflammation Research, Ghent, Belgium.
[5] Department of Applied Mathematics, Computer Science and Statistics, Ghent University, Ghent, Belgium.
[6] Discovery Sciences, Janssen Research & Development, Pharmaceutical Companies of Johnson & Johnson, Beerse, Belgium.
[7] Myeloid Cell Immunology Lab, VIB Center for Inflammation Research, Brussels, Belgium
[8] Laboratory for Molecular and Cellular Therapy, Vrije Universiteit Brussel, Brussels, Belgium

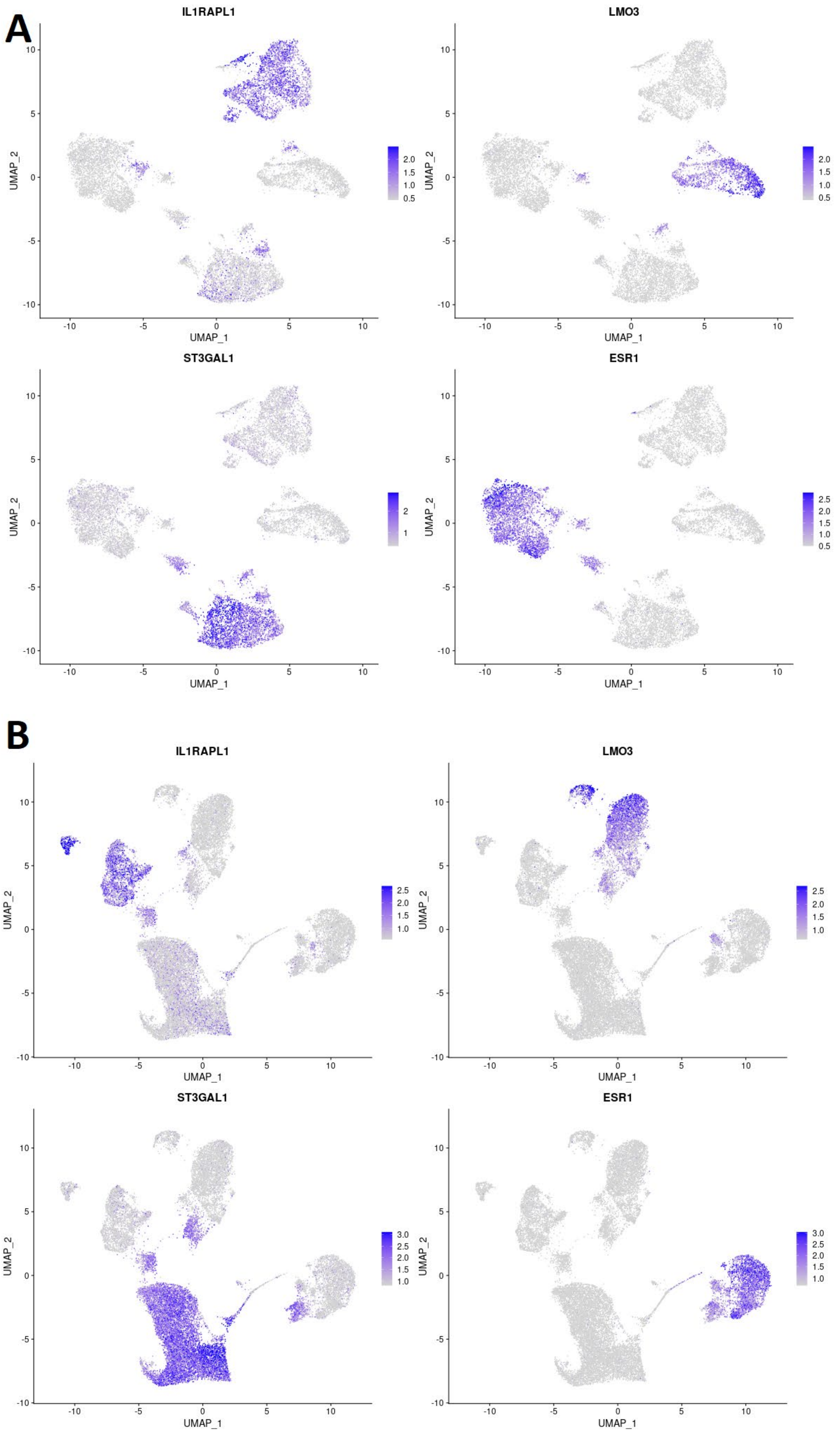

Figure S1. Marker gene expression in TotalSeq-A (A) and custom lipid (B) cell hashing samples. Gene-cell matrices were generated using CellRanger, followed by log-transformation of gene UMI counts and cell clustering (gene expression, PCA reduction) using Seurat. The marker gene UMI counts were visualised in blue color on the gene expression UMAP plots.

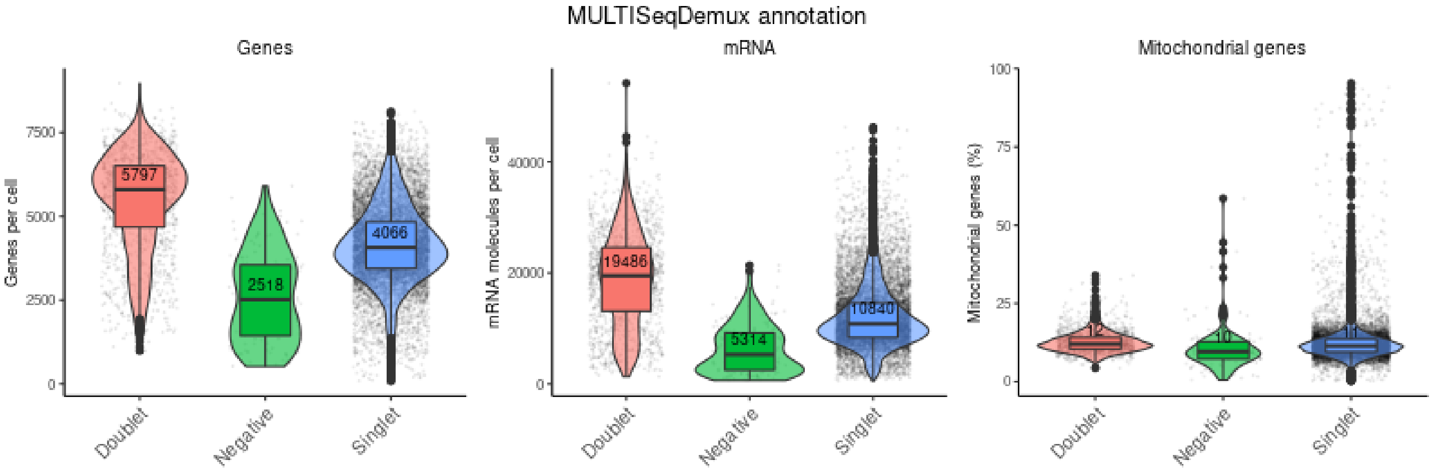

Figure S2. Comparison of MULTISeqDemux-annotated doublets, singlets and negatives. Gene expression were log-transformed using Seurat and detected genes (left plot), UMIs (middle plot) and percentage of mitochondrial genes expression (right plot) in cells were visualised as violin-box plots with median values highlighted in red, across MULTISeqDemux-annotated groups (singlets, doublets, negatives on basis of hashtag expression).

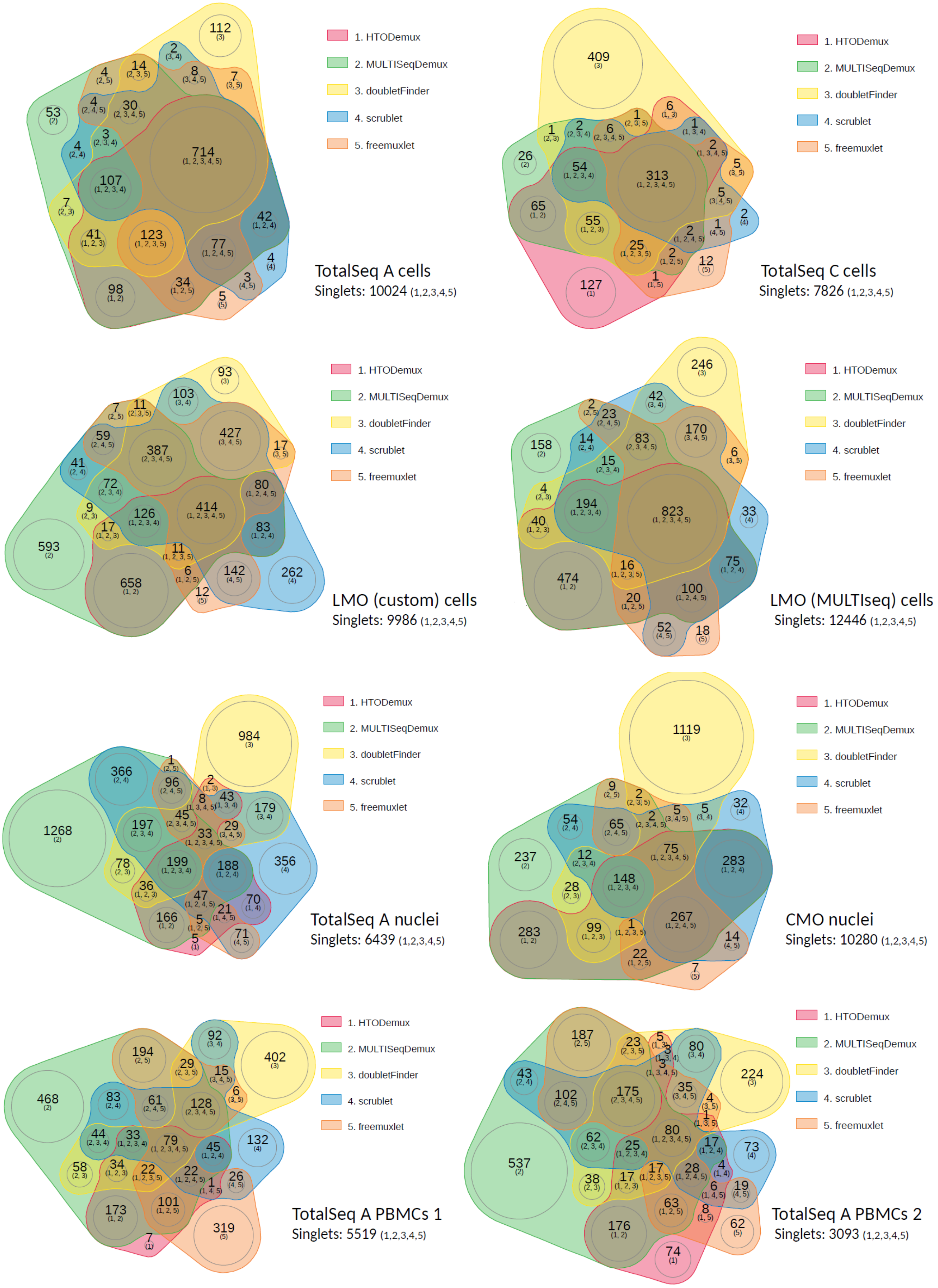

Figure S3. Number of detected doublets by different doublet annotation tools depicted as venndiagrams (“nVennR” package). In parenthesis – which tools compared. For a comparison, number of singlets detected by all 5 tools (1,2,3,4,5) is also shown for each experiment (in the corner).

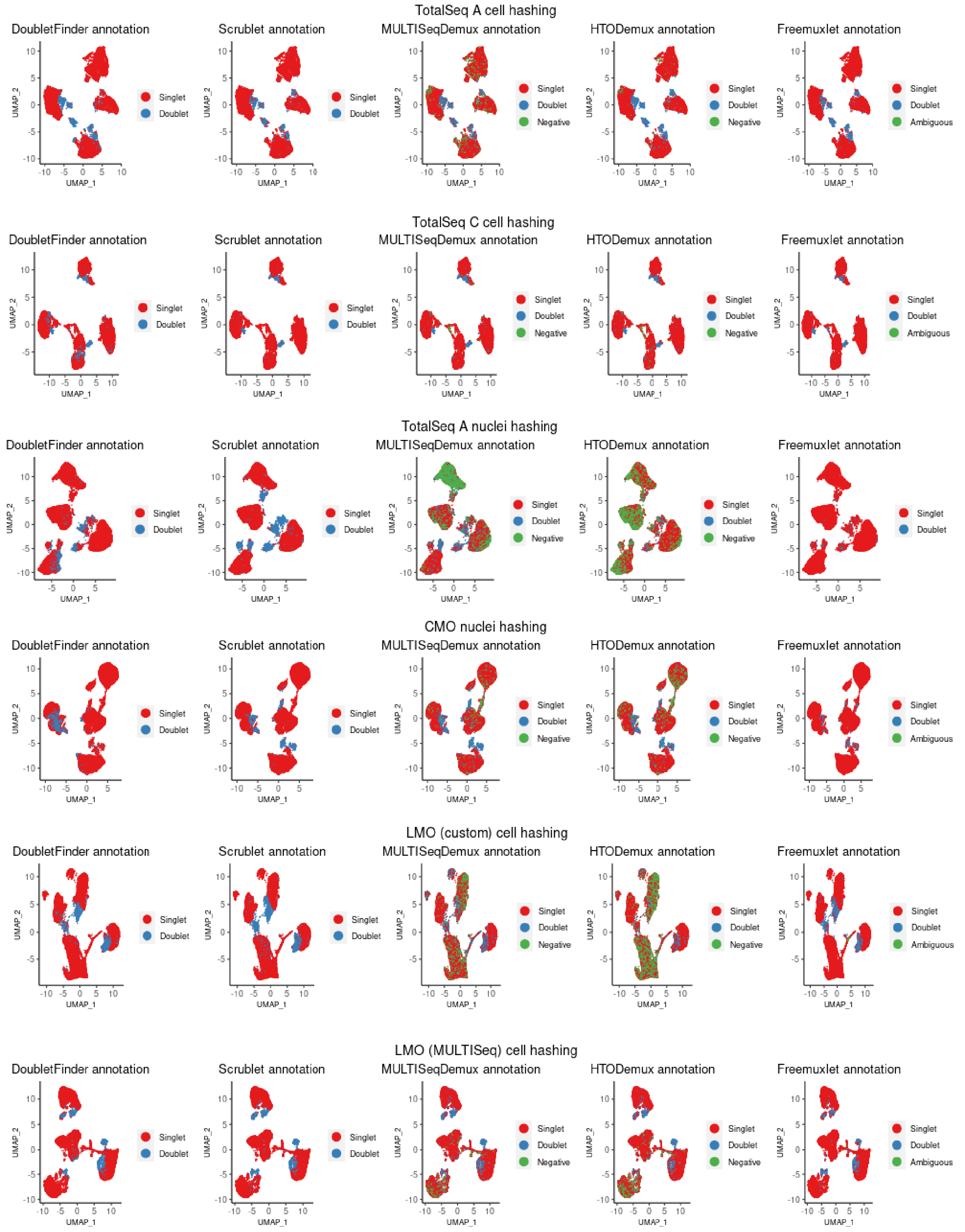

Figure S4. Comparison of doublet annotations (4 cancer cell lines). Gene-cell matrices were generated using CellRanger v3, followed by log-transformation of gene UMI counts and cell clustering (gene expression, PCA reduction) using Seurat. Droplet annotation using 5 different methods is depicted on the gene expression UMAP plots. MULTISeqDemux and HTODemux are the functions from Seurat.

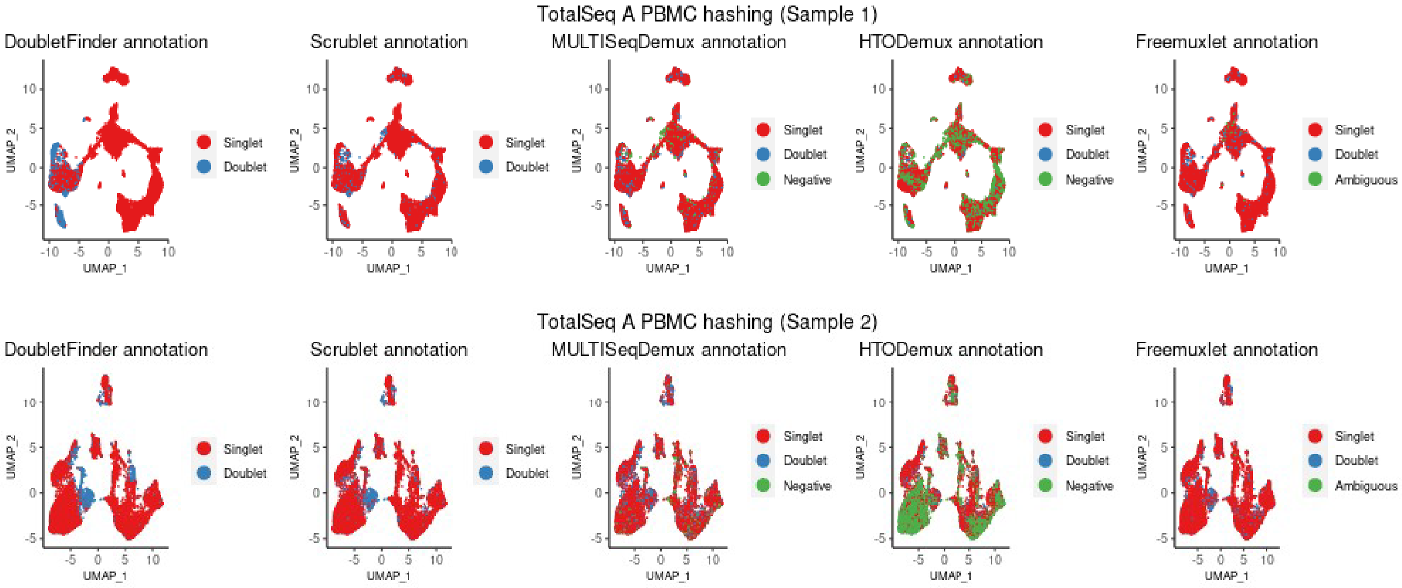

Figure S5. Comparison of doublet annotations (PBMCs). Gene-cell matrices were generated using CellRanger v3, followed by log-transformation of gene UMI counts and cell clustering (gene expression, PCA reduction) using Seurat. Droplet annotation using 5 different methods is depicted on the gene expression UMAP plots. MULTISeqDemux and HTODemux are the functions from Seurat. Sample 1 – healthy patients. Sample 2 – COVID-19 patients.

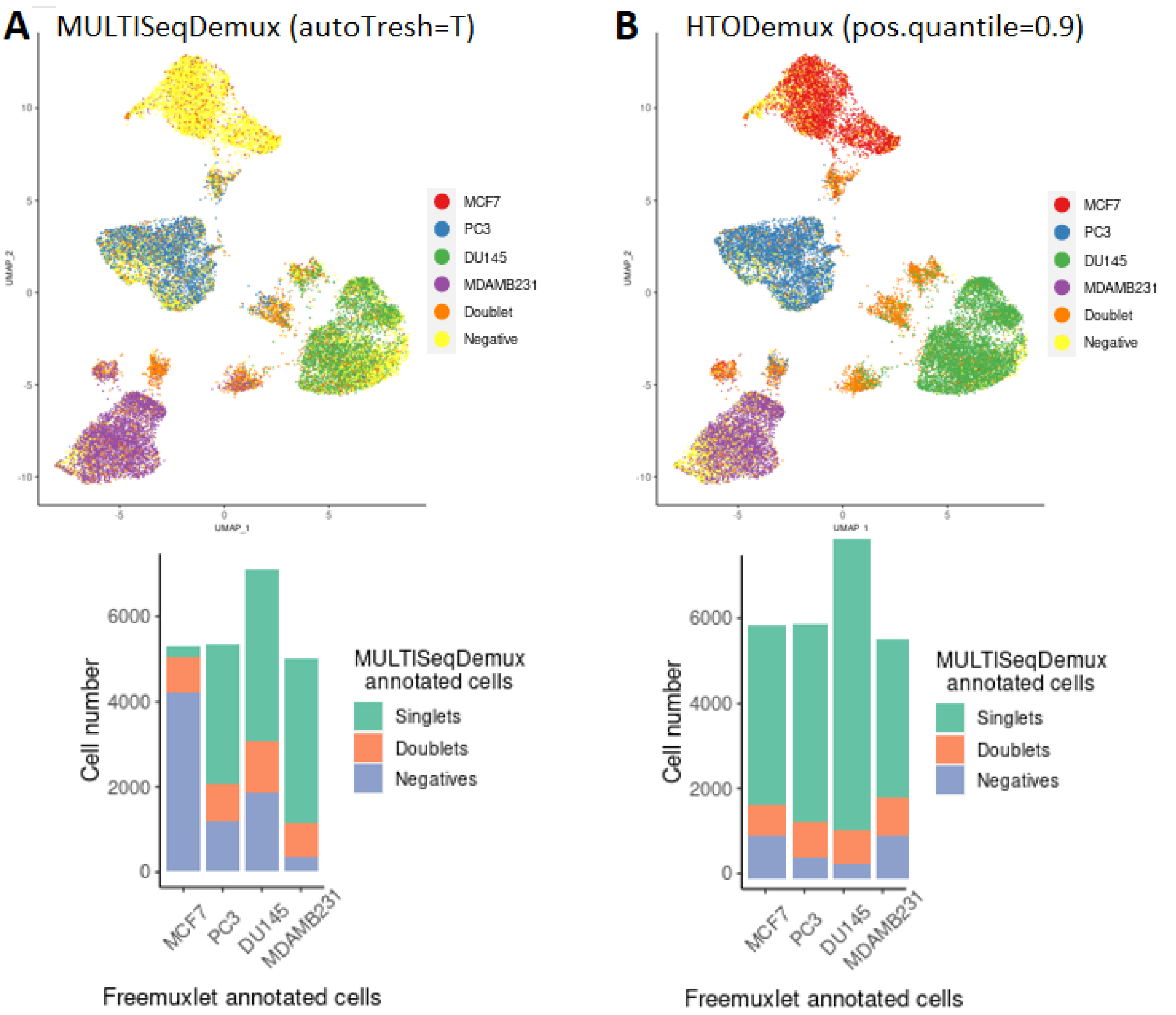

Figure S6. Finetuning demultiplexing. MULTISeqDemux (autoTresh=T) annotation (**A**) vs HTODemux (pos.quant.=0,9) (**B**) on TotalSeq-A nuclei hashing sample. Nuclei annotation (4 cell lines) was performed using freemuxlet (gene expression) or Seurat (MULTISeqDemux function applied on hashtag counts data) and visualized on the gene expression UMAP plots. For the barplots above, MULTISeqDemux-annotated singlets (MCF7, PC3, DU145 or MDAMB231) and negatives (cells with background expression for each hashtag) were matched with the freemuxlet-based annotation (MCF7, PC3, DU145 or MDAMB231). The rest (unmatched) of freemuxlet-annotated singlets were assigned as doublets and altogether visualized as barplots.

A

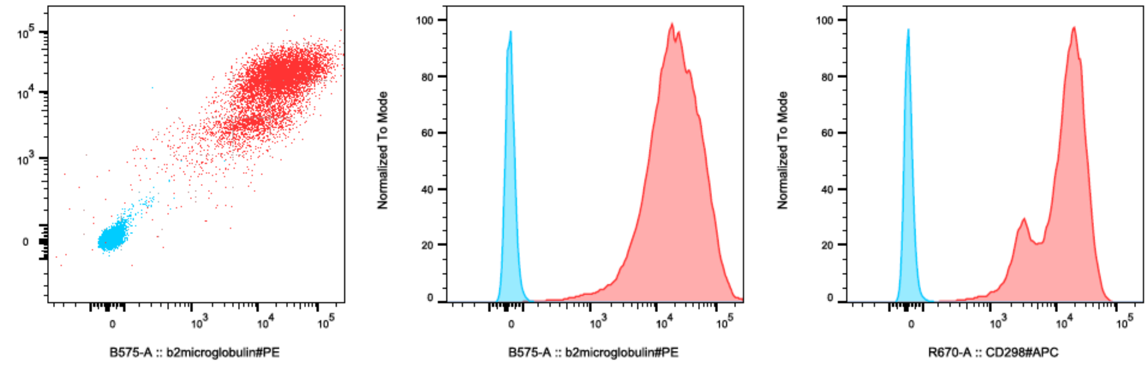

	Sample Name	Subset Name	Count	PE-pos :: Freq. of Parent	APC-pos :: Freq. of Parent
(red)	PBMCs	cells	40549	96.5%	98.2%
(blue)	no abs PBMCs	cells	39514	5.06E-3%	0.020%

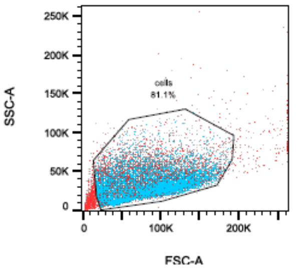

B

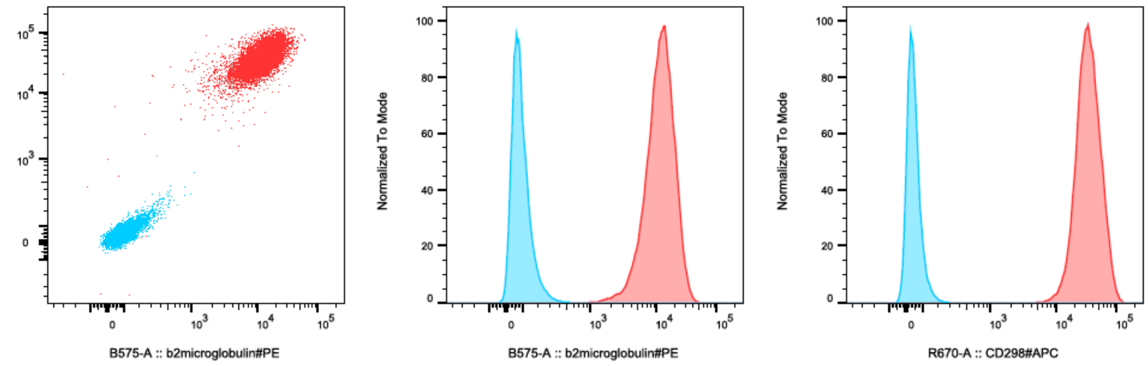

	Sample Name	Subset Name	Count	PE-pos :: Freq. of Parent	APC-pos :: Freq. of Parent
(red)	HEK293A	cells	37391	98.8%	99.9%
(blue)	no abs HEK293A	cells	38519	0%	0%

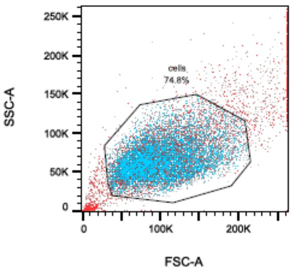

C

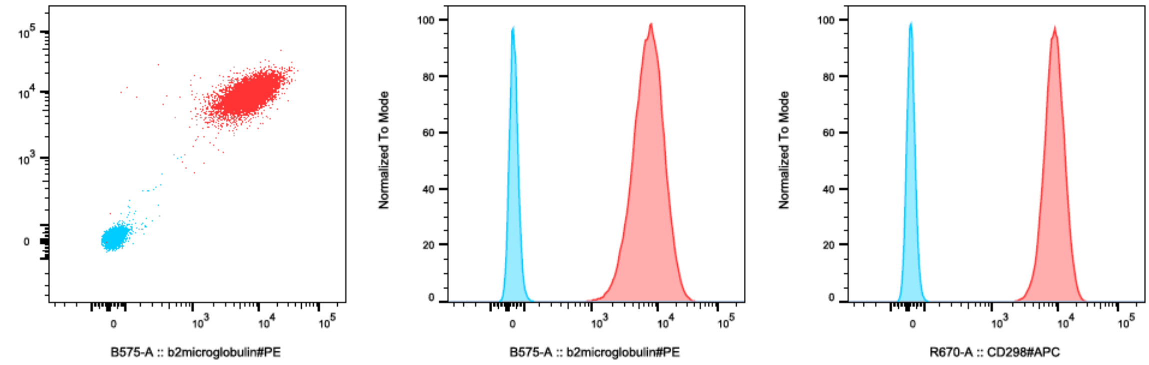

	Sample Name	Subset Name	Count	PE-pos :: Freq. of Parent	APC-pos :: Freq. of Parent
(red)	THP-1	cells	35441	97.9%	100.0%
(blue)	no abs THP-1	cells	37489	0%	0.032%

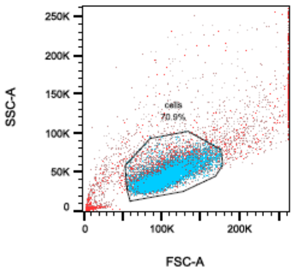

Figure S7. Expression of hashing antigens on human PBMCs (**A**), HEK293A (**B**) and THP-1 (**C**) cells detected by flow cytometry using the same CD298 and b2-microglobulin clones as in the human hashing TotalSeq antibodies. Red color – cells with the antibody staining; blue color – cells without the staining (negative control). Other cells that express both antigens: HeLa, Jurkat, 501-mel, MDA-MB-231, A375m, HIBCPP, HaCaT, BLM, OVCAR-3, HT-29, human fibrosarcoma cells, ARPI9, CAOV-3, HCT116.

A

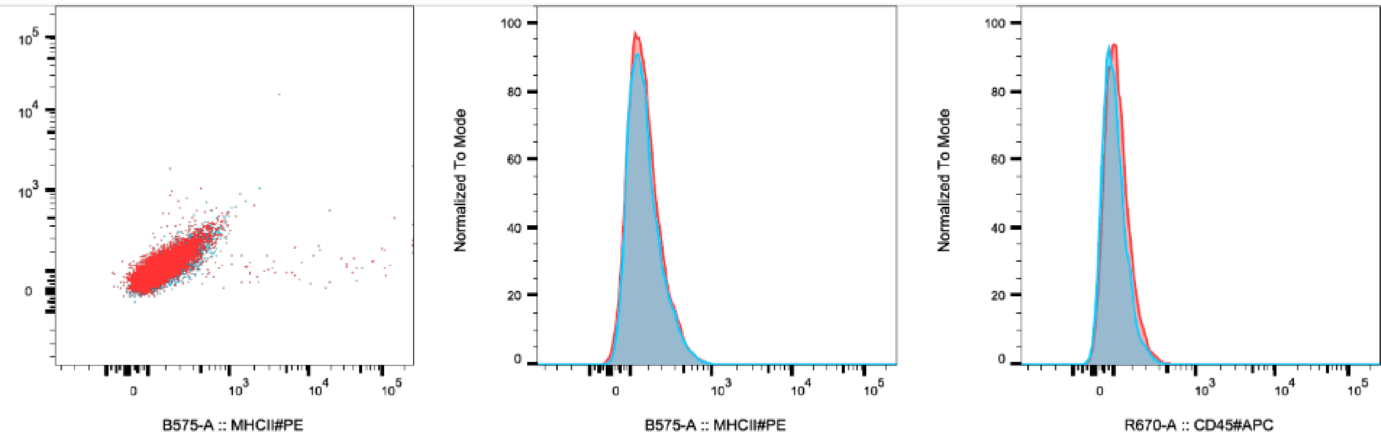

	Sample Name	Subset Name	Count	PE-pos :: Freq. of Parent	APC-pos :: Freq. of Parent
(red)	C3 melanoma	cells	22571	0.34%	0.049%
(blue)	no abs C3	cells	5353	0.037%	0.019%

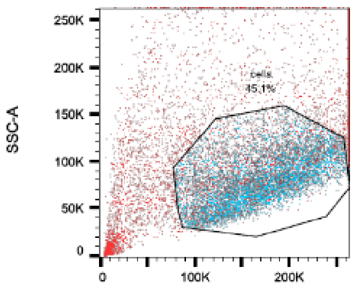

B

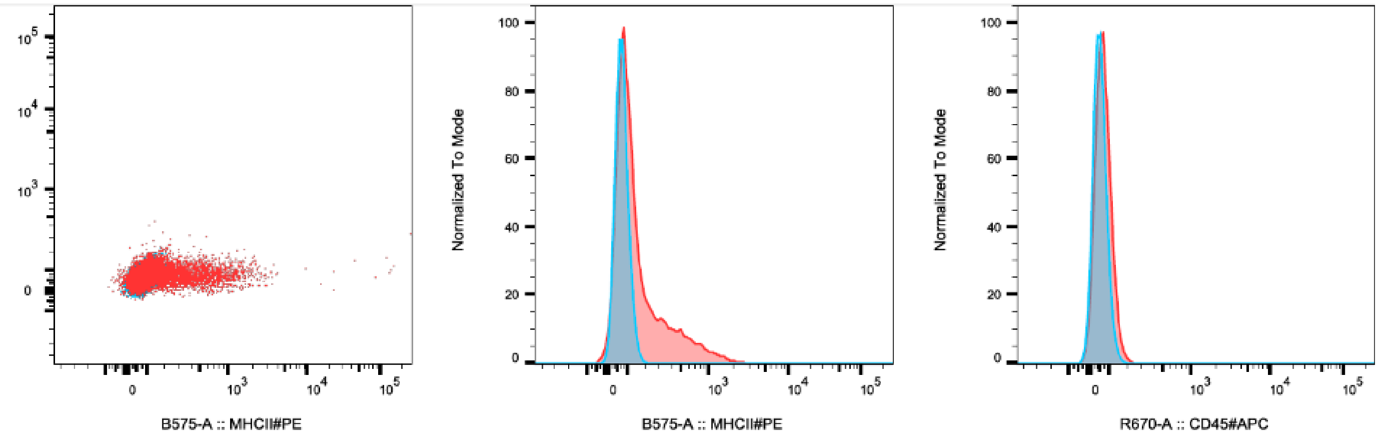

	Sample Name	Subset Name	Count	PE-pos :: Freq. of Parent	APC-pos :: Freq. of Parent
(red)	B16-BL6 melanoma	cells	37765	0.47%	0%
(blue)	no abs B16-BL6	cells	39859	2.51E-3%	0%

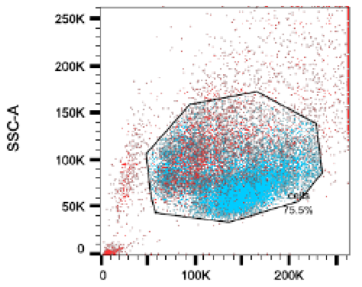

Figure S8. Expression of hashing antigens (CD45 and MHC I) on mouse C3 (**A**) and BL6 melanoma (**B**) cells detected by flow cytometry (same antibody clones as in mouse hashing TotalSeq antibodies). Red color – cells with the antibody staining; blue color – cells without the staining (negative control). The only mouse cell type that expressed both antigens (CD45 and MHC I) from the 8 tested mouse cell lines was J774A1 (macrophages).

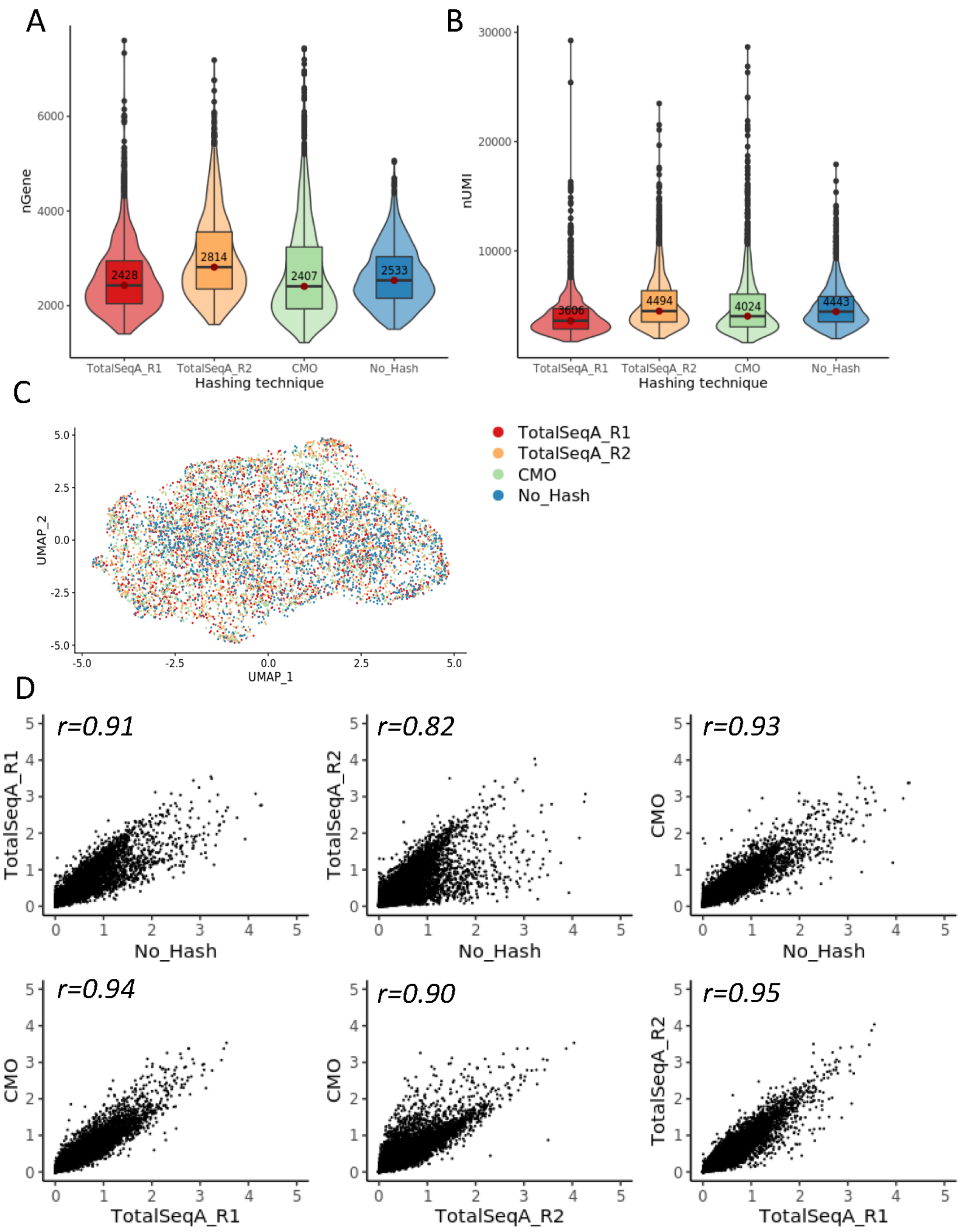

Figure S9. Similarity measures of the gene expression on MCF7 nuclei across the hashing strategies. Gene-nuclei matrices were generated using CellRanger v.3.1, followed by log-transformation of gene UMI counts and nuclei clustering (gene expression, PCA reduction) using Seurat. The hashtag UMI counts were CLR-transformed. To correct the effect of differences in sequencing depth per sample, the reads for each method were down-sampled (DropletUtilis), to match the protocol with the lowest number of total reads (TotalSeq-A_rep2). To the hashtags demultiplexed Seurat objects freemuxlet and DoubletFinder results were added as metadata. For each hashed sample the MCF7 nuclei were extracted from the Seurat object by filtering for i) nuclei with a valid MCF7 hashtag; ii) nuclei assigned by freemuxlet as MCF7 genotype and iii) nuclei assigned as singlets by DoubletFinder. The unhashed sample was processed in the same manner except for filtering for MCF7 hashtags. Each MCF7 nuclei subset was downsized to the hashing method with lowest number of MCF7 nuclei (TotalSeq-A_rep2 2726 cells) and filtered for low quality nuclei (low number of genes per nucleus and high % of mitochondrial genes). Next, the gene expression across the different hashing technologies were normalized, cell cycle and mitochondrial genes were regressed and data were integrated using the SCTransform workflow and **A** detected genes, **B** UMIs in MCF7 nuclei were visualised as violin-box plots with median values highlighted. **C**. The UMAP visualizations on the integrated gene expression. Nuclei are colored by hashing technology. **D.** Average log (gene expression) scatter plots across the hashing technologies on down-sampled data, r= Pearson's correlation.

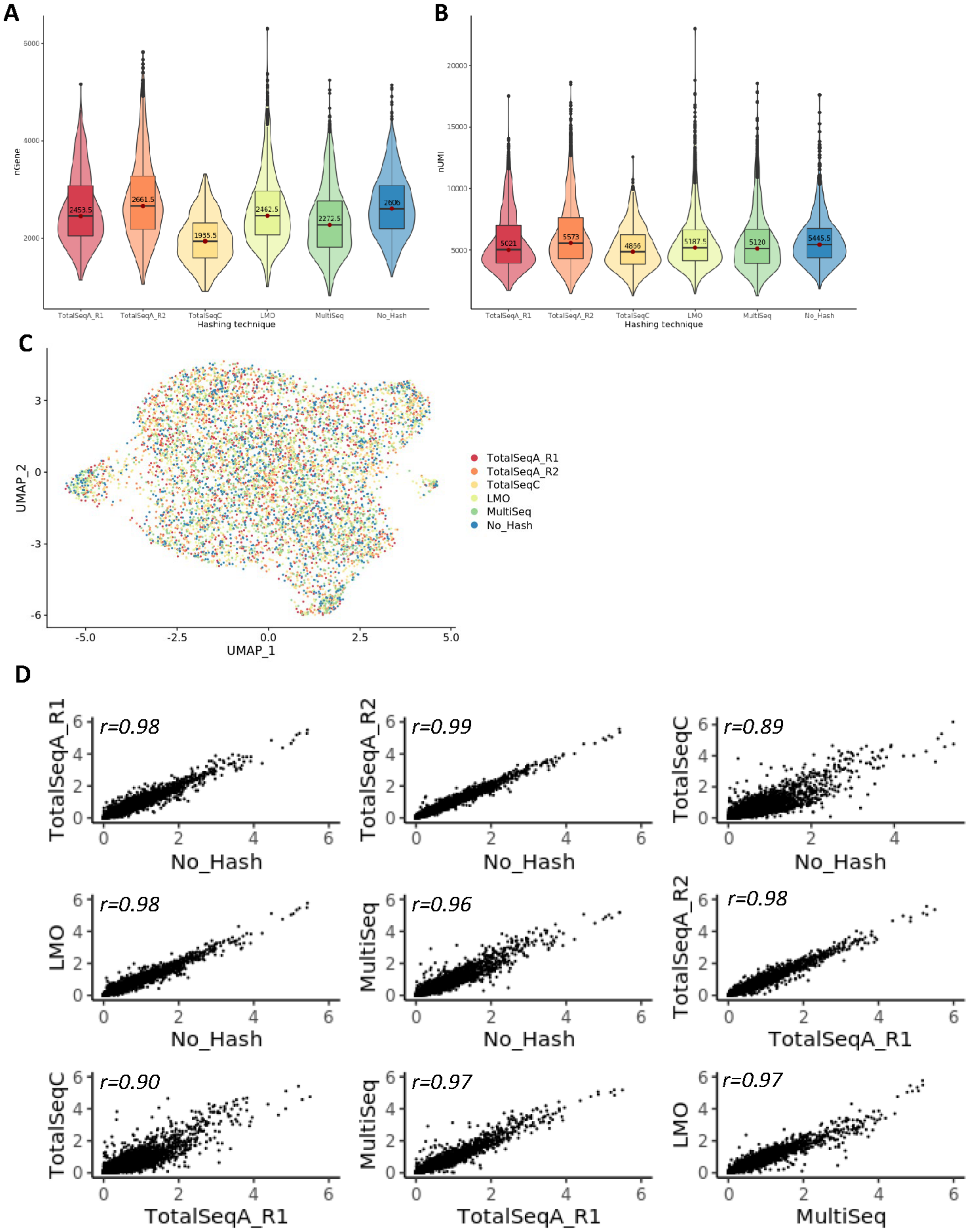

Figure S10. Similarity measures of the gene expression on MCF7 cells across the hashing strategies. Gene-cell matrices were generated using CellRanger v.3.1, followed by log-transformation of gene UMI counts and cell clustering (gene expression, PCA reduction) using Seurat. The hashtag UMI counts were CLR-transformed. To correct the effect of differences in sequencing depth per sample, the reads for each method were down-sampled (DropletUtilis), to match the protocol with the lowest number of total reads (TotalSeq-C). To the hashtag demultiplexed Seurat objects freemuxlet and DoubletFinder results were added as metadata. For each hashed sample the MCF7 cells were extracted from the Seurat object by filtering for i) cells with a valid MCF7 hashtag; ii) cells assigned by freemuxlet as MCF7 genotype and iii) cells assigned as singlets by DoubletFinder. The unhashed sample was processed in the same manner except for filtering for MCF7 hashtags. Each MCF7 cells subset was downsized to the hashing method with lowest number of MCF7 cells (TotalSeq-C 1178 cells) and filtered for low quality cells (low number of genes per cell and high % of mitochondrial genes). Next, the gene expression across the different hashing technologies were normalized, cell cycle and mitochondrial genes were regressed and data were integrated using the SCTransform workflow and **A** detected genes, **B** UMIs in MCF7 cells were visualised as violin-box plots with median values highlighted. **C**. The UMAP visualizations on the integrated gene expression. Cells are colored by hashing technology. **D.** Average log (gene expression) scatter plots across the hashing technologies on down-sampled data (5000), r= Pearson's correlation.

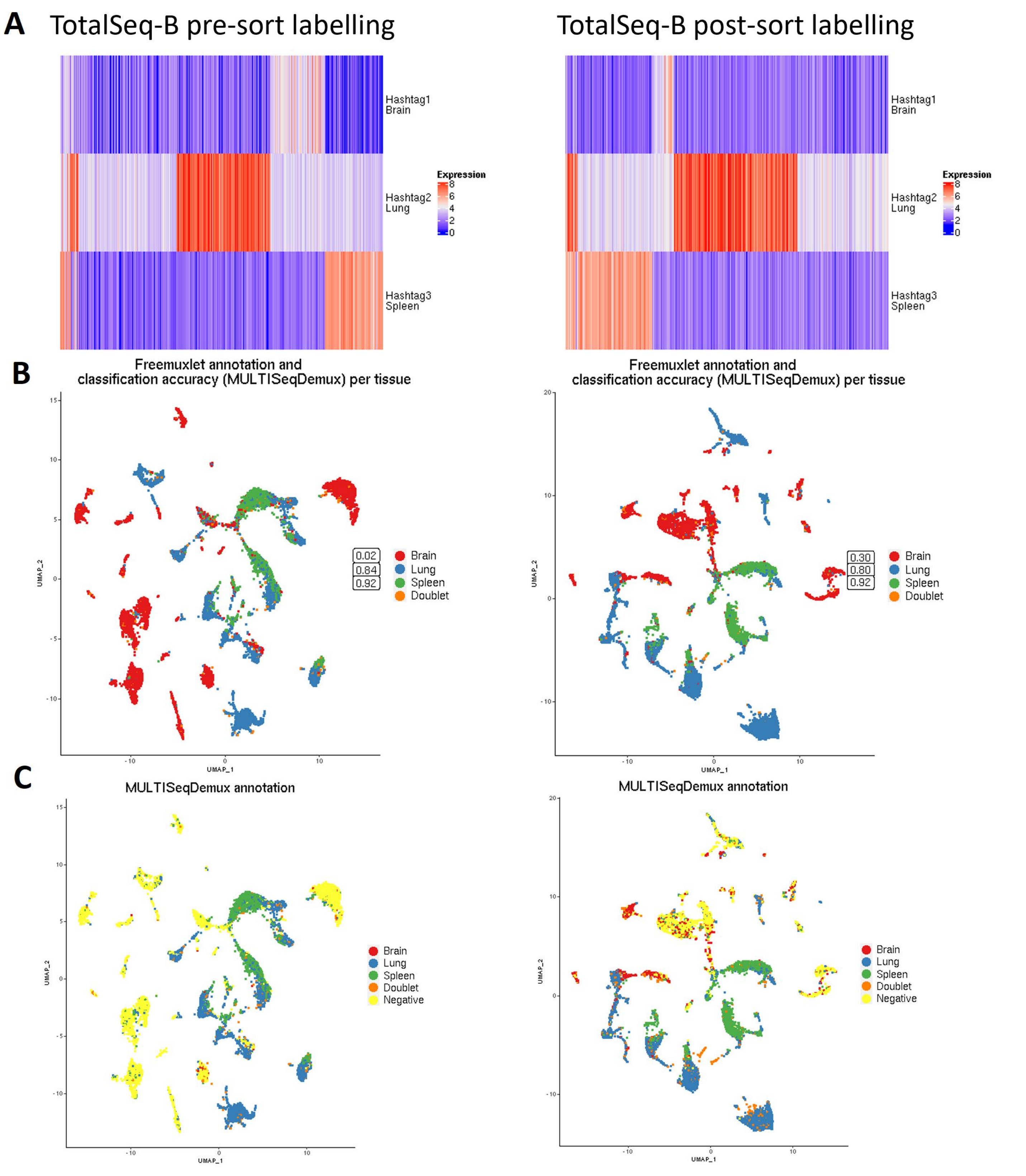

Figure S11. Mice brain, spleen, lung and skin cell antibody hashing with two labelling protocols. Each column represents a separate hashing method. "Pre-sort labelling" – labelling with hashing reagents followed by one wash and live/dead sorting with subsequent loading of the cells on a 10x Genomics chip. "Post-sort labelling" – applying hashing on live-sorted cells followed by 2-3 washes and subsequent loading on a 10x Genomics chip. **A.** Hashtag-derived oligo (HTO) matrices were generated using CellRanger, followed by log-transformation and visualised on heatmaps. **B.** Cell annotation (4 mice strains) was performed using freemuxlet (gene expression) and visualized on the gene expression UMAP plots. Classification accuracy (MULTISeqDemux) of every hashing method reported for each tissue. **C.** MULTISeqDemux-annotated cells (HTO signal) were visualized on the gene expression UMAP plots.

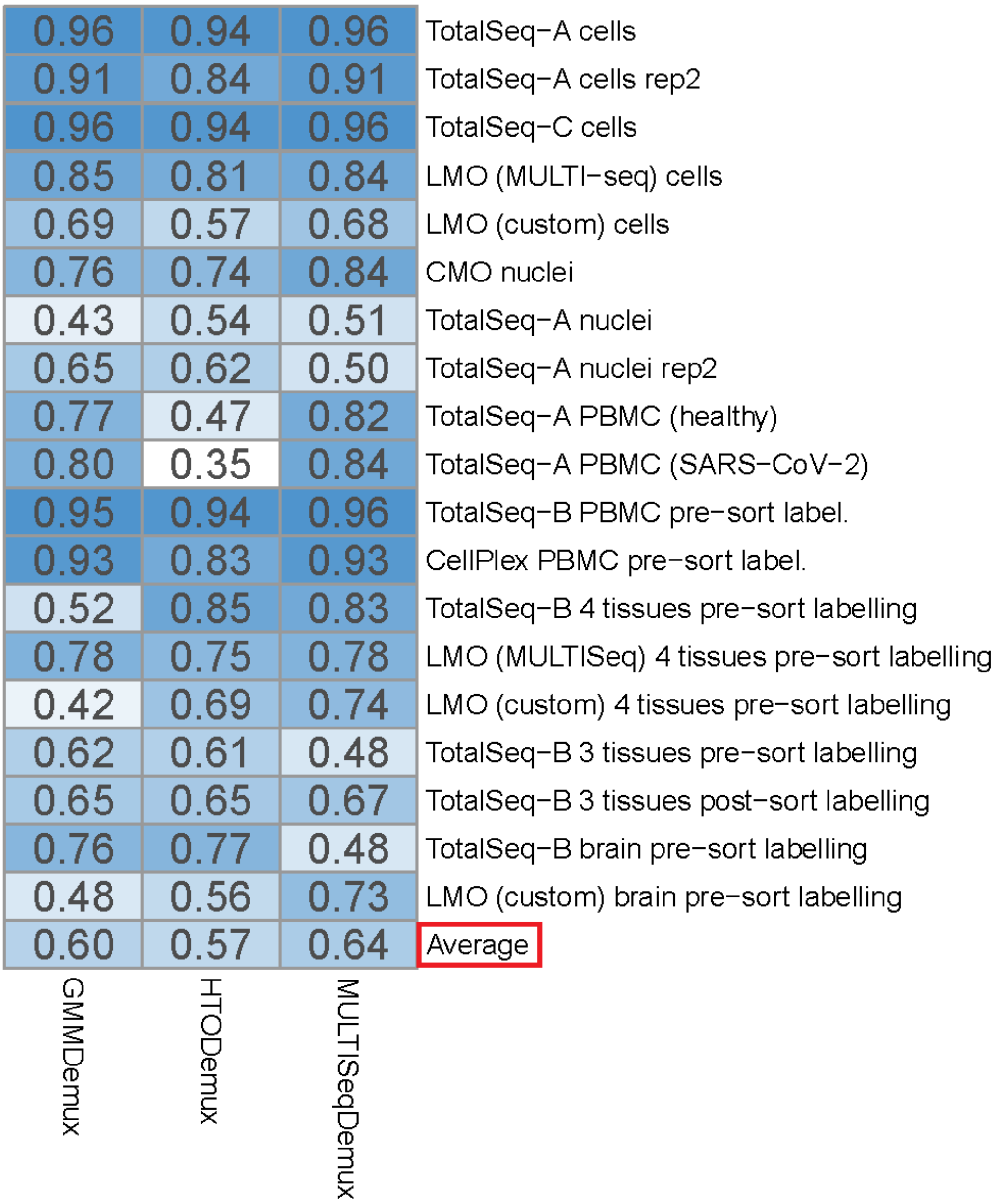

Figure S12. Classification accuracy of MULTISeqDemux, HTODemux and GMMDemux for all datasets. Overall classification accuracy (OCA) for all tested conditions and demultiplexed functions was calculated using freemuxlet demultiplexing as ground truth.

Table S1. **Comparison of doublets detected by freemuxlet and negatives detected by MULTISeqDemux:** Theoretical multiplet rate is based on assumption of 57% cell recovery and equals ~4.6e-06 * number of loaded cells (https://satijalab.org/costpercell/)

Experiment	Estimated Number of Cells	Hashing accuracy (MULTISeq Demux)	Number of doublets (freemuxlet)	Theoretical multiplet number	Number of MULTISeq-annotated singlets among freemuxlet doublets	% of MULTISeq-annotated singlets among freemuxlet doublets	Number of MULTISeq-annotated negatives
1. TotalSeq-A cells	11869	0.96	1031	1137	25	2.42 %	132
2. TotalSeq-A cells rep2	17611	0.91	2325	2503	111	4.77 %	516
3. TotalSeq-C cells	9229	0.96	413	687	26	6.29 %	165
4. LMO (MULTI-seq) cells	16827	0.84	1326	2285	253	19.07 %	1230
5. LMO (custom) cells	21813	0.68	2052	3840	777	37.86 %	4542
6. CMO nuclei	15404	0.84	543	1915	38	6.99 %	1253
7. TotalSeq-A nuclei	23451	0.51%	550	4438	182	33.09 %	7729
8. TotalSeq-A nuclei rep2	9868	0.50	8	786	3	37.5 %	1698
9. TotalSeq-A PBMC1 (healthy)	14635	0.82	1248	1728	521	41.74 %	754
10. TotalSeq-A PBMC2 (SARS-CoV-2)	11372	0.84	837	1044	154	18.39 %	721

Table S2. **Mislabelling ratios**. The mislabelling rate was calculated for each cell line by comparing the cells labelled as singlets by their specific hashtag (MULTISeqDemux) against their genotype (freemuxlet). The labelled cells belonging to a different genotype were considered mislabelled. The mislabelling % is presented as average values +/- SD across the 4 cell lines: MCF7, PC3, DU145, MDAMB231 (or 3 patients for PBMC samples).

Hashing experiment	Mislabeling	SD
1. TotalSeq-A cells	0.1%	± 0.03
2. TotalSeq-A cells rep2	0.18%	± 0.15
3. TotalSeq-C cells	0.11%	± 0.13
4. LMO (MULTI-seq) cells	0.89%	± 1.1
5. LMO (custom) cells	2.67%	± 1.15
6. CMO nuclei	0.12%	± 0.05
7. TotalSeq-A nuclei	4.07%	± 2.51
8. TotalSeq-A nuclei rep2	3.81%	± 4.24
9. TotalSeq-A PBMC1 (healthy)	4.76%	± 1.93
10. TotalSeq-A PBMC2 (SARS-CoV-2)	1.53%	± 1.06

Table S3. TotalSeq-A anti-nucleoporin antibody barcode sequences:

Hashtag	Antibody barcode
1.	A0458 TGACGCCGTTGTTGT
2.	A0459 GCCTAGTATGATCCA
3.	A0456 CTCGAACGCTTATCG
4.	A0457 CTTATCACCGCTCAA

Table S4. LMOs, CMOs and sample barcode oligonucleotides:

Custom LMO Anchor:	5’-AGTGACAGCTGGATCGTTAC[Palmitate]-3’
Custom LMO Co-anchor:	5’-[Stearyl]GTAACGATCCAGCTGTCACTCACGTCTGAACTCCAGTCAC-3’
CMO Anchor:	5’AGTGACAGCTGGATCGTTAC[Chol-TEG]-3’
LMO Co-anchor:	5’[Chol-TEG]GTAACGATCCAGCTGTCACTCACGTCTGAACTCCAGTCAC-3’
Hashtag 1	TTGTCACGGTAATTA
Hashtag 2	ATCGAACCGACAGAG
Hashtag 3	GGTCGAATATGTCGG
Hashtag 4	CTCAAGCATTATCAT
