# Supplementary material for: Comparative analysis of antibody- and lipid-based multiplexing methods for single-cell RNA-seq
Source: Genome Biol. 2022 Feb 16;23:55. doi: 10.1186/s13059-022-02628-8 (PMC8851857; doi:10.1186/s13059-022-02628-8)
Supplement: Supplementary file 2 — Additional file 2. Reviewer History. [file 13059_2022_2628_MOESM2_ESM.docx]

Review History

**First round of review**

**Reviewer 1**

**Are you able to assess all statistics in the manuscript, including the appropriateness of statistical tests used?**

No, I do not feel adequately qualified to assess the statistics.

**Comments to author:**

Summary

In their manuscript, Mylka and colleagues performed a comparison of different hashing methods for cells and nuclei. Through mixtures of genetically distinct cell lines the authors demonstrate antibodies are superior for intact cells and lipids are superior for nuclei.

Sample multiplexing is widely used by the single-cell genomics community and is performed by many laboratories around the world without strong guidelines on their use.

A comparative study of these different hashing methods has not been reported in the scientific literature before and is of broad interest to the field. Despite the title of the manuscript being "Comparative analysis of antibody- and lipid-based multiplexing methods for single-cell RNA-seq" a comprehensive benchmarking study is lacking from the authors. They present data from experiments with a limited diversity of samples and omit some commercially available hashing reagents. I would suggest performing additional experiments or narrowing the claims of the manuscript.

Major points:

1. The authors perform most experiments with human cancer cell line mixtures using a limited number of samples. Although useful as a sample for benchmarking experiments due to their genetically diverse nature they do not represent the typical sample used in single-cell RNA-seq experiments. Apart from a minor supplementary figure, mouse cells are not used in comparative experiments. In agreement with the authors, in our experience hashtag antibodies work robustly in human samples but inconsistently in mouse samples. A formal demonstration of this would be important for the community.

a. Aside from human PBMCs where only 1 reagent was used, tissues which contain a diverse array of cell types, are not presented in the manuscript.

b. I suggest the authors perform a hashtag comparison experiment in a well characterised mouse tissue sample, such as brain or intestine. A mixture of different mouse strains or distinct tissues could be used to estimate ground truth.

c. It would also be informative to investigate how both TotalSeq and Multiseq compare as the number of samples is increased (e.g from 4 to 8). Also mixing

2. The authors compare total-seq A and total-seq C antibodies. Total-seq C is officially supported by 10x Genomics while total-seq A is not. Due to differences in molecular biology total-seq B may be superior to total-seq A for sample hashtagging. This is an important comparison to make for a comprehensive benchmarking study.

3. The effects of differences in sample preparation for nuclei and cell suspensions, labelling protocols, sequencing library preparation has not been explored. While the number of all possible modifications is vast and is certainly beyond the scope of this work, reducing the comparison to a single "one-size fits all" protocol per method is somewhat limited. Especially relevant in this context is robustness of the protocols, and if (and how) standard protocols can/should be adjusted to a specific tissue or cell type.

Minor points:

Introduction and Results

1. Line 53: Can the authors clarify why chemical labelling with oligos is challenging and was not performed in this study?

2. Line 83: I prefer the term "level of detection" as opposed to "level of expression". Expression to me indicates a molecule produced by the cell which is not applicable to lipid hashtags.

3. Line 99: Where comparisons of genes and UMIs are made can the authors indicate if a statistical test was performed?

4. Table 1: For a fair comparison downsampling to fixed number of hashtag reads per cell should be performed.

5. Line 151: Where comparisons are made between different hashing methods it would be clearer to the reader for results to be shown side by side in a histogram visualisation.

6. Line 179: Should be Figure 6C vs 7C.

7. Line 204: Cell clustering by transcriptome indicates all cell types were detected in the PBMC samples. "Data not shown". In my experience there is a minor difference in total-seq hashtag labelling between immune subsets. Can this data be shown with hashtag detection overlaid?

8. Line 240: The signal to noise ratio has been shown to strongly depend on the ratio of hashtag reagent to cell number Buus, T. B., et al., (2020). Improving oligo-conjugated antibody signal in multimodal single-cell analysis BioxRiv https://doi.org/doi.org/10.1101/2020.06.15.153080. I recommend the authors discuss their results from Supp figure 2 in light of these findings.

Methods

9. Line 315: The recommended amount of Total-seq antibody is 1ug. Can the authors clarify why 10 fold lower antibody was used? Similar comment for anti-nucleus antibody.

10. Line 374: Indicate the amount of hashtag antibody used in the PBMC experiment.

11. Line 375: Indicate if the 3 samples pooled together were all heathy donors or SARS-CoV-2 patients. Are the heathy donors and SARS-CoV-2 patients 2 independent experiments?

Figures and tables

12. Annotations although legible are on the small side. Recommend enlarging the font for all figures.

13. CLR transformation obscures the sample dependent background of hashtag labelling. A useful visualisation would be a heatmap of log transformed, un-normalised counts to enable an assessment of signal to background of each hashtag method.

14. Figure 9B: Please comment on why patient mixing is better for heathy controls than for SARS-CoV-2 PBMCs.

15. Supplemental table 2: A column indicating theoretical doublets based on number of cells loaded would be useful.

16. Supplemental figure 4 would be more concise as a series of barplots or similar with different classification algorithm results on the same plot.

17. Figure 2 to Figure 9 follow exactly the same pattern, the authors could make this more interesting.

18. Figure 1 could be condensed.

Conclusion

The authors have produced a well controlled study and guidelines for the use case of human cell lines labelled with antibodies or lipids. However a comprehensive benchmarking study requires mouse cells and tissue samples.

**Reviewer 2**

**Are you able to assess all statistics in the manuscript, including the appropriateness of statistical tests used?**

Yes, and I have assessed the statistics in my report.

**Comments to author:**

In this manuscript, the authors conducted a comparative study to test the efficiency of antibody- and lipid-based multiplexing methods for scRNA-seq data. Indeed, it has many attractive features to pool samples together for single cell sequencing, and it is a hot research topic for sample demultiplexing and doublet identification. Specifically, the authors use samples from 4 cell lines (both scRNA-seq and single-nucleus RNA-seq) as well as clinical PBMC samples from 3 healthy and 3 SARS-Cov-2 infected patients to compare the performance of different cell hashing experiments and different computational pipelines. They found that antibody-based cell hashing is most efficient for cells and lipid-based hashing is better for nuclei. I have some comments below:

Major comments:

1. Although the results may provide valuable information to researches who are deciding the hashing strategy, the study lacks of novelty and has limited scope. The tests were done mostly on cell lines which lack complexity of types of cells. It would be more interesting to see data from biological more relevant samples such as lung, skin, or intestine tissues. It is also of importance to test hashing efficiency in PBMC in a cell type specific manner.

2. In line 57-59, the authors state that they use demultiplexing by genetic diversity to build "ground truth". The authors should elaborate more on this, for example, how accurate is the result from demultiplexing by genetic diversity through the tool freemuxlet so that it can be used as a reference annotation to assess the efficiency of cell hashing. Also, do the authors think cell hashing should only be used when demultiplexing by genetic diversity is not applicable (i.e., no genetic variance among samples), since the latter is considered as "ground truth".

3. In line 72-76, the authors define the hashing efficiency as the proportion of freemuxlet-annotated ("ground truth") singlets being successfully identified as singlets by Seurat, and this measurement is considered as one of the deciding factors to draw conclusions. As in a binary classification problem (singlet as 0 and doublet as 1), this measurement is essentially the same as specificity. In my opinion, it is a better approach to look at both specificity and sensitivity, since I don't see a reason why specificity is more important than sensitivity here. For example, the hashing efficiency defined here can achieve 100% if Seurat identifies all cells as singlets, which apparently doesn't represent a "good result". I would suggest the users use summary measures such as Youden Index and/or overall classification accuracy (i.e., (True Positive + True Negative)/total N) to assess the cell hashing efficiency.

4. In this manuscript, the authors applied two functions for demultiplexing cell hashing data, MULTISeqDemux and HTODemux. I suggest the authors include another method, GMM-Demux (https://doi.org/10.1186/s13059-020-02084-2), since it is a newly developed method for sample demultiplexing with cell hashing data and published on Genome Biology last year.

5. Section Antibody hashing can be successfully applied on clinical PBMC samples (line 196-213) is too brief. Since the authors mentioned they have performed clustering analysis, they should elaborate more on this part. For example, if there are any cell clusters that are formed by cell doublets, and if demultiplexing by cell hashing or by genetic diversity fail to identify any of such cell clusters.

6. In addition to comparing different methods with data from four samples (cell lines), I suggest the authors increase the number of samples to eight to ten to see if the same conclusions still hold.

7. On lines 60-61 in the Introduction: "Single-cell suspensions can only be prepared from fresh tissues, which is a major roadblock for analysing clinical samples". We and others have successfully analyzed cells isolated from small pieces of some tissues that were previously cryopreserved.

Minor comments:

1. In line 86-87, the authors state cells with more than one HTO generally group together in between the larger clusters. However, it is not very straightforward to observe those cells in Figure 2A. I suggest the authors should directly highlight cells with positive expressions of multiple HTOs on one UMAP plot so that people can easily observe those cells.

2. Supplemental Figure 3 is hard to read. The authors could try to add the performance of each method by comparing the result with the ground truth.

3. Have the authors check the performance of nuclei hashing antibody by FACS?

4. Minor: Sup. Fig. 8. Expression of hashing antigens (CD45 and MHC II), this should be MHC I

**Authors Response**

**Point-by-point responses to the reviewers’ comments:**

Reviewer reports:

Reviewer #1: Summary

In their manuscript, Mylka and colleagues performed a comparison of different hashing methods for cells and nuclei. Through mixtures of genetically distinct cell lines the authors demonstrate antibodies are superior for intact cells and lipids are superior for nuclei.

Sample multiplexing is widely used by the single-cell genomics community and is performed by many laboratories around the world without strong guidelines on their use.

A comparative study of these different hashing methods has not been reported in the scientific literature before and is of broad interest to the field. Despite the title of the manuscript being "Comparative analysis of antibody- and lipid-based multiplexing methods for single-cell RNA-seq" a comprehensive benchmarking study is lacking from the authors. They present data from experiments with a limited diversity of samples and omit some commercially available hashing reagents. I would suggest performing additional experiments or narrowing the claims of the manuscript.

*We would like to thank the reviewer for giving objective feedback and comments regarding our manuscript. It definitely helped to improve the paper.*

Major points:

1. The authors perform most experiments with human cancer cell line mixtures using a limited number of samples. Although useful as a sample for benchmarking experiments due to their genetically diverse nature they do not represent the typical sample used in single-cell RNA-seq experiments. Apart from a minor supplementary figure, mouse cells are not used in comparative experiments. In agreement with the authors, in our experience hashtag antibodies work robustly in human samples but inconsistently in mouse samples. A formal demonstration of this would be important for the community.

a. Aside from human PBMCs where only 1 reagent was used, tissues which contain a diverse array of cell types, are not presented in the manuscript. b. I suggest the authors perform a hashtag comparison experiment in a well characterised mouse tissue sample, such as brain or intestine. A mixture of different mouse strains or distinct tissues could be used to estimate ground truth.

*Thank you for these comments. We acknowledge that addressing hashing on mouse primary tissues is extremely important and quite challenging at the same time. During the revision we performed and included into the paper a number of big hashing experiments. Two different cell hashing experiments with almost the same set-up (thus 2 replicates in total): cell pools from mouse brain, lung and spleen (3 different strains), plus skin in one of the experiments; pre-sorting and post-sorting labelling protocols; and using 1 or 2 lipid types and TotalSeq B antibodies. We also added data from another experiment with a different brain cell isolation protocol (3 mice strains) that involved antibody and lipid hashing.*

*As a result, we added a new part “Antibody and lipid hashing of primary mouse tissues” and new figures (Fig. 5, 6 and sup.fig. 11) to the paper.*

c. It would also be informative to investigate how both TotalSeq and Multiseq compare as the number of samples is increased (e.g from 4 to 8). Also mixing 2.

*Thank you for the comment. We believe that the comparison of different sample numbers is rather a theoretical problem. As was already shown by Satija lab and 10x Genomics (Fig. 1), increasing the number of hashed samples increases the number of detectable multiplets.*

*Fig.1 (10x Genomics webpage)*

*Because the total multiplet rate depends only on the number of cells loaded, the ability to distinguish true singlets from homotypic multiplets increases linearly when increasing the number of hashes. E.g. when 17500 cells are loaded (2 hashes), there will be 733 multiplets that are non-identifiable; 490 when 3 hashes are used; 374 when 4 hashes, and only 189 when 8 hashes are used (https://satijalab.org/costpercell/). At the same time the specific binding capacity of hashes that defines the signal-to-noise ratio should not depend on the number of samples, especially taken into account that the samples are pooled immediately before chip loading. Therefore we decided to focus on addressing other issues, e.g. mouse hashing etc., and kept using 3-4 hashed and pooled samples, as this number is often used by single cell users due to an optimal cell loading limit for 10x kits.*

The authors compare total-seq A and total-seq C antibodies. Total-seq C is officially supported by 10x Genomics while total-seq A is not. Due to differences in molecular biology total-seq B may be superior to total-seq A for sample hashtagging. This is an important comparison to make for a comprehensive benchmarking study.

*TotalSeq-B and -C antibodies indeed share the same principle: unique capture sequences that don’t compete for binding with mRNA, unlike the TotalSeq-A that have a polyA capture sequence. Nevertheless, we know from our work on CITE-seq optimisation, that this competition does not impact significantly the number of detected genes and UMIs per cell when comparing samples with TotalSeq-A antibodies and without (mock CITE-seq). We also know that all 3 types of antibodies have comparable abilities to detect major cell markers, and proteins detected by TotalSeq-B and -C antibodies have very similar expression profiles (Fig 2 below). For example this is prominent in the case of the HLA-DR protein which belongs to the MHC-II molecules (Fig.2 below, second row from the bottom). The closely related hashing antigen beta-microglobulin belongs to MHC-I. Thus, we decided to evaluate TotalSeq-B antibodies not on the cell lines but on PBMCs using another protocol where HTO reads per cell were downsampled to match the TotalSeq-A PBMCs samples (a different labelling protocol though). We also switched to TotalSeq-B antibodies in our mouse tissue experiments.*

*Fig.2. Samples with TotalSeq A, -B and -C antibodies demonstrate comparable profiles of normalised UMIs (violinplot) for the majority of the markers. 3000 of raw antibody reads and 37 000 raw gene reads per cell for each sample were subsampled from fastq files. Afterwards gene-cell matrices were created with cellranger 3 following the reads mapping to hg19 genome. Then, 2000 cells were subsetted from corresponding Seurat objects followed by antibody UMI normalisation using centered log-ratio (CLR) transformation transformation and visualised as the violinplots.*

3. The effects of differences in sample preparation for nuclei and cell suspensions, labelling protocols, sequencing library preparation has not been explored. While the number of all possible modifications is vast and is certainly beyond the scope of this work, reducing the comparison to a single "one-size fits all" protocol per method is somewhat limited. Especially relevant in this context is robustness of the protocols, and if (and how) standard protocols can/should be adjusted to a specific tissue or cell type.

A*ll the parameters mentioned above by the reviewer are without doubt very important for the output of hashing experiments. Indeed addressing all of these would require a massive undertaking. With the available manpower, budget and time we managed to address the following during the revision:*

*- Comparison of TotalSeq-B hashing with custom and MULTISeq lipids on mice brain, lung, skin and spleen cells.*

*- comparison of labelling protocols for mice brain, lung and spleen cells: pre-sort hashtag labelling vs post-sort labelling;*

*- testing of 2 protocols for mouse brain cells isolation;*

*- we also compared demultiplexing with freemuxlet of cells from closely related mice strains (e.g. C57BL6/N vs C57BL6/J) with more distinct strains (C57BL6 vs BALB/C)*

*Also we added to the paper:*

*- TotalSeq-B vs CellPlex hashing on human PBMCs using a different labelling strategy (“pre-sort labelling”)*

*The appropriate section (“Antibody and lipid hashing of primary mouse tissues”) and figures (Fig. 5,6 and Sup.fig.11) were added to the Results section. Also the Discussion part was rewritten to reflect these additional experiments.*

*We tried to address the following, but unfortunately in contrast to regular snRNAseq, the quality of RNA and/or hashing accuracy of brain nuclei were very low:*

*- comparison of mouse brain nuclei antibody hashing (Gaublomme et al labelling protocol) with CMO (MULTISeq protocol) nuclei hashing (3 different mice strains).*

*- comparison of Frankenstein and TST protocol for mouse brain nuclei isolation followed by antibody and lipid hashing.*

*We did several repeats of the brain nuclei hashing. Among a few optimisations, we applied centrifugation recommendations from the Gaublomme et al. nuclei hashing paper. We even added an excess of RNAse inhibitor to the labelling buffer in the second repeat (RNAse inhibitor is not present in the labelling buffer from the above-mentioned paper). We also executed a completely new experiment where we used fresh brains for nuclei isolation and received the same low quality of the RNA and very low hashing accuracy. In the previous trials frozen brains were used. We are not adding this part to the story, because we did not figure out why the hashing didn’t work here.*

*The appropriate section (“Antibody and lipid hashing of primary mouse tissues”) and figures (Fig. 5,6 and Sup.fig.11) are added to the Results section. Also the Discussion part is rewritten.*

Minor points:

Introduction and Results

1. Line 53: Can the authors clarify why chemical labelling with oligos is challenging and was not performed in this study?

*In our single trial here, we could not generate any HTO (hashtag-derived oligo) cDNA, as indicated by the result of the QC using BioAnalyzer. Thus we decided to focus on other hashing methods.*

*We replaced “tend to be” with “can be” in the text: “…chemical labelling with oligos can be more challenging.”*

2. Line 83: I prefer the term "level of detection" as opposed to "level of expression". Expression to me indicates a molecule produced by the cell which is not applicable to lipid hashtags.

*Thank you for this suggestion, we adapted this in the manuscript.*

3. Line 99: Where comparisons of genes and UMIs are made can the authors indicate if a statistical test was performed?

*Thank you for the suggestions, we now added this information to the manuscript.*

4. Table 1: For a fair comparison downsampling to fixed number of hashtag reads per cell should be performed.

*All the samples are now normalized at the level of FASTQ HTO reads as indicated in Figure 1.*

5. Line 151: Where comparisons are made between different hashing methods it would be clearer to the reader for results to be shown side by side in a histogram visualisation.

*A new figure (Figure 1) is added instead of the tables 1 and 2.*

6. Line 179: Should be Figure 6C vs 7C.

*Thank you. This has been adapted.*

7. Line 204: Cell clustering by transcriptome indicates all cell types were detected in the PBMC samples. "Data not shown". In my experience there is a minor difference in total-seq hashtag labelling between immune subsets. Can this data be shown with hashtag detection overlaid?

*Thank you for the suggestions. A new figure (Figure 4C ) was added with hashing accuracy per each major cell type +/- SD representing variation between 3 donors. Also the percentage of mitochondrial genes per each cell type was added.*

8. Line 240: The signal to noise ratio has been shown to strongly depend on the ratio of hashtag reagent to cell number Buus, T. B., et al., (2020). Improving oligo-conjugated antibody signal in multimodal single-cell analysis BioxRiv https://doi.org/doi.org/10.1101/2020.06.15.153080. I recommend the authors discuss their results from Supp figure 2 in light of these findings.

*We added more clarifications to the Discussion part:” The impaired doublet annotation due to increased multiplet rate together with a failure to assign hashtags to a number of cells (“negatives”) might explain the lower hashing efficiency in the samples with higher number of cells loaded/captured on chip. For example, the captured cell number in the repeated TotalSeq-A experiment (rep2) was increased by 48%, while the number of doublets (detected by freemuxlet) and negatives (assigned by MULTISeqDemux) increased by 125% and 325%, respectively. An important parameter , the “signal-to-noise ratio” (Buus et al.) , was kept in this comparison on the same level by labelling equal number of cells with the same quantity of hashing antibodies.” Thus in both experiments we labelled 200 000 cells with 0,1 ug of antibodies followed by loading of ~16 000 cells in one experiment and ~50 000 cells in the “superloading “ experiment. Subsequently the increased multiplet rate (especially for non-identifiable or homotypic multiplets) is the main driver of decreased hashing accuracy in the “superloaded” sample.*

Methods

9. Line 315: The recommended amount of Total-seq antibody is 1ug. Can the authors clarify why 10 fold lower antibody was used? Similar comment for anti-nucleus antibody.

*As explained in the comment above, we tried to keep the signal-to-noise ratio on a similar level, at least per category studied (e.g. cells or nuclei). We based our calculation on the CITE-seq protocol from NYGC: https://citeseq.files.wordpress.com/2019/02/cell_hashing_protocol_190213.pdf*

*where ~1-2 M cells are labelled with 0,5 ug of antibody. We were labelling 200 000 cells (to mimic samples with limited number of cells), thus chose 5 fold less antibodies. This proportion was also based on our CITE-seq optimisation work. For nuclei hashing we opted for 0,5 ug because we were labelling ~ 850 000 nuclei.*

10. Line 374: Indicate the amount of hashtag antibody used in the PBMC experiment.

*This info is added now. As a part of the clinical trial, samples here were labelled with 0.025 ug of antibody but in smaller volume- 25 ul. Also we want to mention that the main focus of this trial was on the CITE-seq part (not included in this hashing paper though).*

11. Line 375: Indicate if the 3 samples pooled together were all heathy donors or SARS-CoV-2 patients. Are the heathy donors and SARS-CoV-2 patients 2 independent experiments?

*We added the clarification: “…pooled together (3 healthy donors resulting in sample one and 3 SARS-CoV-2 patients resulting in sample two)…”*

*Both samples were from one experiment*

Figures and tables

12. Annotations although legible are on the small side. Recommend enlarging the font for all figures.

*Thank you, we increased the font size now.*

13. CLR transformation obscures the sample dependent background of hashtag labelling. A useful visualisation would be a heatmap of log transformed, un-normalised counts to enable an assessment of signal to background of each hashtag method.

*Thank you for this suggestion. We followed it and replaced all the CLR-transformed UMAP plots with heatmaps of log-transformed, unnormalised hashtag counts in all figures.*

14. Figure 9B: Please comment on why patient mixing is better for heathy controls than for SARS-CoV-2 PBMCs.

*In the day of the experiment dozens of samples were processed without cell counting before loading onto 10x chip due to time restriction. Thus the SARS-CoV-2 samples probably had higher variation in terms of cell viability.*

15. Supplemental table 2: A column indicating theoretical doublets based on number of cells loaded would be useful.

*Thank you , we now added this to the table*

16. Supplemental figure 4 would be more concise as a series of barplots or similar with different classification algorithm results on the same plot.

*This visualisation is indeed not concise, but we believe that the UMAP plots are more informative than barplots in this context. For instance two methods might predict the same number of doublets, but they will be from different group of cells, thus easier noticeable when having “spatial” visualisation when using UMAP plots.*

17. Figure 2 to Figure 9 follow exactly the same pattern, the authors could make this more interesting. 18. Figure 1 could be condensed.

*We hope we managed to improve this. We changed layout and re-grouped all the figures. (Figures 2-6)*

Conclusion

The authors have produced a well controlled study and guidelines for the use case of human cell lines labelled with antibodies or lipids. However a comprehensive benchmarking study requires mouse cells and tissue samples.

Reviewer #2: In this manuscript, the authors conducted a comparative study to test the efficiency of antibody- and lipid-based multiplexing methods for scRNA-seq data. Indeed, it has many attractive features to pool samples together for single cell sequencing, and it is a hot research topic for sample demultiplexing and doublet identification. Specifically, the authors use samples from 4 cell lines (both scRNA-seq and single-nucleus RNA-seq) as well as clinical PBMC samples from 3 healthy and 3 SARS-Cov-2 infected patients to compare the performance of different cell hashing experiments and different computational pipelines. They found that antibody-based cell hashing is most efficient for cells and lipid-based hashing is better for nuclei. I have some comments below:

*We are grateful for this review and for all the comments that helped us to improve our manuscript.*

Major comments:

1. Although the results may provide valuable information to researches who are deciding the hashing strategy, the study lacks of novelty and has limited scope. The tests were done mostly on cell lines which lack complexity of types of cells. It would be more interesting to see data from biological more relevant samples such as lung, skin, or intestine tissues. It is also of importance to test hashing efficiency in PBMC in a cell type specific manner.

*We address the following during the revision:*

*- Comparison of TotalSeq B hashing with custom and MULTISeq lipids on mice brain, lung, skin and spleen cells.*

*- comparison of labelling protocols for mice brain, lung and spleen cells: pre-sort hashtag labelling vs post-sort labelling;*

*- testing of 2 protocols for mouse brain cells isolation;*

*- we also compared demultiplexing with freemuxlet of cells from closely related mice strains (e.g. C57BL6/N vs C57BL6/J) with more distinct strains (C57BL6 vs BALB/C)*

*-*

*Also we added to the paper:*

*TotalSeq-B vs CellPlex hashing on human PBMCs using a different labelling strategy (“pre-sort labelling”)*

*The appropriate section (“Antibody and lipid hashing of primary mouse tissues”) and figures (Fig. 5,6 and Sup.fig.11) were added to the Results section. Also the Discussion part was rewritten to reflect these additional experiments.*

*We tried to address the following, but unfortunately in contrast to regular snRNAseq, the quality of RNA and/or hashing accuracy of brain nuclei were very low:*

*- comparison of mouse brain nuclei antibody hashing (Gaublomme et al labelling protocol) with CMO (MULTISeq protocol) nuclei hashing (3 different mice strains).*

*- comparison of Frankenstein and TST protocol for mouse brain nuclei isolation followed by antibody and lipid hashing.*

*We did several repeats of the brain nuclei hashing. Among a few optimisations, we applied centrifugation recommendations from the Gaublomme et al. nuclei hashing paper. We even added an excess of RNAse inhibitor to the labelling buffer in the second repeat (RNAse inhibitor is not present in the labelling buffer from the above-mentioned paper). We also executed a completely new experiment where we used fresh brains for nuclei isolation and received the same low quality of the RNA and very low hashing accuracy. In the previous trials frozen brains were used. We are not adding this part to the story, because we did not figure out why the hashing didn’t work here.*

*The appropriate section (“Antibody and lipid hashing of primary mouse tissues”) and figures (Fig. 5,6 and Sup.fig.11) are added to the Results section. Also the Discussion part is rewritten.*

*Regarding the PBMC cell types hashing accuracy, a new figure (Figure 4C) was added with hashing accuracy per each major cell type +/- SD representing variation across 3 donors.*

2. In line 57-59, the authors state that they use demultiplexing by genetic diversity to build "ground truth". The authors should elaborate more on this, for example, how accurate is the result from demultiplexing by genetic diversity through the tool freemuxlet so that it can be used as a reference annotation to assess the efficiency of cell hashing. Also, do the authors think cell hashing should only be used when demultiplexing by genetic diversity is not applicable (i.e., no genetic variance among samples), since the latter is considered as "ground truth".?

*Freemuxlet (https://github.com/statgen/popscle) is an open-source software tool where we have extensive experience with, but to our knowledge there are no benchmarks available on the performance of this tool. We have conducted the following in-house test for the performance of this tool. We combined the scRNAseq data of two bronchoalveolar lavage samples from two male unrelated individuals from the same study. In a first step unique sample identifiers were added to the cell barcodes to introduce the ground truth. Next the files were merged in to one BAM file that was used as input for freemuxlet. The results are visualized in the table below, with over 99% of all cells correctly classified as singlets.*

*Based on our results we now added the following sentence to the Dicussion “). Although it is possible to demultiplex samples using only freemuxlet when pooling cells from different patients or from distinct mouse strains, the tool fails to separate strains such as C57BL6N and C57BL6J for example.” Additionally we observed and report the following “…on the TotalSeq-A cell hashing sample, MULTISeqDemux demonstrated 0.94 OCA (overall classification accuracy) when compared with freemuxlet, and 0.95 when compared with demuxlet as a reference”. Just as a reminder, demuxlet requires external SNP reference information for demultiplexing.*

3. In line 72-76, the authors define the hashing efficiency as the proportion of freemuxlet-annotated ("ground truth") singlets being successfully identified as singlets by Seurat, and this measurement is considered as one of the deciding factors to draw conclusions. As in a binary classification problem (singlet as 0 and doublet as 1), this measurement is essentially the same as specificity. In my opinion, it is a better approach to look at both specificity and sensitivity, since I don't see a reason why specificity is more important than sensitivity here. For example, the hashing efficiency defined here can achieve 100% if Seurat identifies all cells as singlets, which apparently doesn't represent a "good result". I would suggest the users use summary measures such as Youden Index and/or overall classification accuracy (i.e., (True Positive + True Negative)/total N) to assess the cell hashing efficiency.

*Thank you for the suggestion. We followed your recommendations and applied for all datasets in this study the Overall Classification Accuracy metrics (i.e., (True Positive + True Negative)/total N). (Figure1)*

4. In this manuscript, the authors applied two functions for demultiplexing cell hashing data, MULTISeqDemux and HTODemux. I suggest the authors include another method, GMM-Demux (https://doi.org/10.1186/s13059-020-02084-2), since it is a newly developed method for sample demultiplexing with cell hashing data and published on Genome Biology last year.

*Thank you for the suggestions. GMM-Demux annotation was now added to all the datasets (Figure 1).*

5. Section Antibody hashing can be successfully applied on clinical PBMC samples (line 196-213) is too brief. Since the authors mentioned they have performed clustering analysis, they should elaborate more on this part. For example, if there are any cell clusters that are formed by cell doublets, and if demultiplexing by cell hashing or by genetic diversity fail to identify any of such cell clusters.

*We added the comparison of hashing accuracy across major cell types (T/NK cells, DCs/monocytes, B cells) for all PBMC samples.*

6. In addition to comparing different methods with data from four samples (cell lines), I suggest the authors increase the number of samples to eight to ten to see if the same conclusions still hold.

*Thank you for the comment. We believe that the comparison of different sample numbers is rather a theoretical problem. As was already shown by Satija lab and 10x Genomics (Fig. 1), increasing the number of hashed samples increases the number of detectable*

*Fig.1 (10x Genomics webpage)*

*multiplets. Because the total multiplet rate depends only on the number of cells loaded, the ability to distinguish true singlets from homotypic multiplets increases linearly when increasing the number of hashes. E.g. when 17500 cells are loaded (2 hashes), there will be 733 multiplets that are non-identifiable; 490 when 3 hashes are used; 374 when 4 hashes, and only 189 when 8 hashes are used (https://satijalab.org/costpercell/). At the same time the specific binding capacity of hashes that defines the signal-to-noise ratio should not depend on the number of samples, especially taken into account that the samples are pooled immediately before chip loading. Therefore we decided to focus on addressing other issues, e.g. mouse hashing etc., and kept using 3-4 hashed and pooled samples, as this number is often used by single cell users due to an optimal cell loading limit for 10x kits.*

7. On lines 60-61 in the Introduction: "Single-cell suspensions can only be prepared from fresh tissues, which is a major roadblock for analysing clinical samples". We and others have successfully analyzed cells isolated from small pieces of some tissues that were previously cryopreserved.

*Actually we have also experienced this at our lab recently, but it can be at the expense of specific cell types. Thus we edited in the text as follows “Single-cell suspensions are most commonly prepared from fresh tissues…”.*

Minor comments:

1. In line 86-87, the authors state cells with more than one HTO generally group together in between the larger clusters. However, it is not very straightforward to observe those cells in Figure 2A. I suggest the authors should directly highlight cells with positive expressions of multiple HTOs on one UMAP plot so that people can easily observe those cells.

*UMAP plots in A panel are replaced with heatmaps now. Multiplets are visualised in panels B and C (Fig.2-6) which have the same cluster visualisations (gene data UMAPs), thus should be easy to compare.*

2. Supplemental Figure 3 is hard to read. The authors could try to add the performance of each method by comparing the result with the ground truth.

*The new OCA metrics throughout the text and all the figures incorporates also multiplet annotation when comparing to “ground truth” (freemuxlet).*

3. Have the authors check the performance of nuclei hashing antibody by FACS?

*No, unfortunately this was not done.*

4. Minor: Sup. Fig. 8. Expression of hashing antigens (CD45 and MHC II), this should be MHC I

*Thank you for spotting this. This is corrected now. Thank you once again for all the valuable comments and suggestions that helped us improve our story.*

**Second round of review**

**Reviewer 2**

In this revised manuscript, authors have addressed most of my comments. They performed additional experiments to compare antibody-based hashing and lipid based hashing on additional tissues including mice brain, lung, skin and spleen cells. They also compared and optimized different protocols. Overall, I am pleased with the extensive response. I have a few additional minor comments.

1. Authors mentioned "MULTISeqDemux function with autoTresh =T parameter originally tailored for lipid-based hashing, performs even better than the HTODemux function, and only slightly better than the GMM-Demux function on a vast majority of tested data sets". It would be better to show the results for each program (e.g. confusion matrix) in Supplemental Figures 3-6, which may provide guidance of the demux program in future studies.

2. The raw data is still not public on ENA. Authors should make it public upon the acceptance of the paper. They should also release all labels from each of the programs (MULTISeqDemux, HTODemux, GMMDemux, and freemuxlet). It will be valuable to bioinformatics field for new method developments.

3. The doublets formed by two cells with the same barcodes (within sample doublets) are still indistinguishable. I wonder if authors can comment on this and discuss if any of the multiplexing methods may help detect or at least estimate such fraction.

**Authors Response**

**Point-by-point responses to the reviewers’ comments:**

Reviewer #2: In this revised manuscript, authors have addressed most of my comments. They performed additional experiments to compare antibody-based hashing and lipid based hashing on additional tissues including mice brain, lung, skin and spleen cells. They also compared and optimized different protocols. Overall, I am pleased with the extensive response. I have a few additional minor comments. 1. Authors mentioned "MULTISeqDemux function with autoTresh =T parameter originally tailored for lipid-based hashing, performs even better than the HTODemux function, and only slightly better than the GMM-Demux function on a vast majority of tested data sets". It would be better to show the results for each program (e.g. confusion matrix) in Supplemental Figures 3-6, which may provide guidance of the demux program in future studies.

*We added new Fig. S12 with classification accuracy of MULTISeqDemux, HTODemux and GMMDemux for all datasets in a single heatmap with all values and an average value for each demultiplexing method.*

2. The raw data is still not public on ENA. Authors should make it public upon the acceptance of the paper. They should also release all labels from each of the programs (MULTISeqDemux, HTODemux, GMMDemux, and freemuxlet). It will be valuable to bioinformatics field for new method developments.

*The datasets (raw fastq files and tables with the labels for each demultiplexing methods) are now publicly available at ENA.*

*https://www.ebi.ac.uk/arrayexpress/experiments/E-MTAB-9964/*

*https://www.ebi.ac.uk/arrayexpress/experiments/E-MTAB-11401/*

3. The doublets formed by two cells with the same barcodes (within sample doublets) are still indistinguishable. I wonder if authors can comment on this and discuss if any of the multiplexing methods may help detect or at least estimate such fraction.

*That’s a very good question and according to our knowledge there is no a single method that can handle this issue with very high precision. Thus we add the following phrasing in the Discussion section to give a bit more clarifications on this issue for the readers and users. New text is in bold:*

*“…This can be partially explained by the reliance on different data modalities that can lead to a certain bias in detection of heterotypic or homotypic doublets. For instance, the functions incorporated in Seurat* ***(e.g. HTO-Demux, MULTISeqDemux)*** *predict doublets based on the hashtag signal* ***(in particular combinations of different hashtags)****, while other tools* ***(e.g. DoubletFinder)****use the gene expression* ***data and can detect doublets formed by transcriptionally-distinct cells. Thus it’s worthwhile to consult both types of methods (gene expression- and HTO-based) while keeping in mind a theoretical multiplet rate (~0.8% for every 1000 captured cells on a 10x Genomics chip, CG000206 Rev D).”***
